# High-resolution HDX-MS reveals distinct mechanisms of RNA recognition and activation by RIG-I and MDA5

**DOI:** 10.1093/nar/gku1329

**Published:** 2014-12-24

**Authors:** Jie Zheng, Hui Yee Yong, Nantika Panutdaporn, Chuanfa Liu, Kai Tang, Dahai Luo

**Affiliations:** 1 School of Biological Sciences, Nanyang Technological University, 60 Nanyang Drive, 637551, Singapore; 2 Lee Kong Chian School of Medicine, Nanyang Technological University, 61 Biopolis Drive, Proteos Building, #07-03, 138673, Singapore

## Abstract

RIG-I and MDA5 are the major intracellular immune receptors that recognize viral RNA species and undergo a series of conformational transitions leading to the activation of the interferon-mediated antiviral response. However, to date, full-length RLRs have resisted crystallographic efforts and a molecular description of their activation pathways remains hypothetical. Here we employ hydrogen/deuterium exchange coupled with mass spectrometry (HDX-MS) to probe the *apo* states of RIG-I and MDA5 and to dissect the molecular details with respect to distinct RNA species recognition, ATP binding and hydrolysis and CARDs activation. We show that human RIG-I maintains an auto-inhibited resting state owing to the intra-molecular HEL2i-CARD2 interactions while *apo* MDA5 lacks the analogous intra-molecular interactions and therefore adopts an extended conformation. Our work demonstrates that RIG-I binds and responds differently to short triphosphorylated RNA and long duplex RNA and that sequential addition of RNA and ATP triggers specific allosteric effects leading to RIG-I CARDs activation. We also present a high-resolution protein surface mapping technique that refines the cooperative oligomerization model of neighboring MDA5 molecules on long duplex RNA. Taken together, our data provide a high-resolution view of RLR activation in solution and offer new evidence for the molecular mechanism of RLR activation.

## INTRODUCTION

In the vertebrate immune system, RIG-I (Retinoic acid inducible gene 1) like receptors (RLRs) are the major cytoplasmic surveillance proteins that defend the host against viral infection by sensing viral RNA (1–3). There are three members of the RLR gene family: RIG-I, MDA5 (melanoma differentiation associated gene 5) and LGP2 (Laboratory of Genetics and Physiology 2). All RLRs contain a conserved RNA sensing module consisting of a helicase domain (HEL) linked to the C-terminal domain (CTD), but only RIG-I and MDA5 contain the signaling module at their N-termini composed of tandem caspase activation and recruitment domains (CARDs; CARD1 and CARD2) (4–7). Therefore, RLR antiviral activity is believed to be mediated mainly by RIG-I and MDA5. The CARDs of RIG-I and MDA5 interact with the common adaptor protein, mitochondrial antiviral-signaling protein (MAVS, also known as IPS-1, VISA or CARDIF), to initiate a downstream signaling cascade and activate the production of type I interferon (IFN) and inflammatory cytokines (6,8–12).

The RNA sensing modules of RIG-I and MDA5 are homologous in sequence and structure. The HEL domain comprises a conserved Rec-A-like-helicase core (HEL1 and HEL2) and a novel insertion domain HEL2i. Because of this unique domain architecture RLRs are classified as double-stranded RNA stimulated ATPases (DRAs), which also include Dicer and Dicer-related helicases (13). DRAs preferentially recognize duplex RNA and play important roles in antiviral defense, RNA interference, regulation of gene activities and genomic integrity (13). The CTD is a zinc ion binding domain that recognizes blunt-ended duplex RNA preferably bearing a 5′ triphosphate group such as defective interfering (DI) viral RNA which are often generated during the viral replication process (14,15). Very recently, RIG-I has also been shown to mediate specific antiviral responses to RNAs bearing 5′-diphosphates which can be found at the termini of the genomes from reoviruses (segmented, double-stranded RNA viruses) (16). RNA recognition by the CTD is mediated by the highly conserved triphosphate-binding pocket and the RNA duplex end-capping loop (17–19). RIG-I has also been shown to be activated by long duplex RNA (polyIC), but the conformation of RIG-I binding internally to long duplex RNA remains elusive (2,20–23). The CTD of MDA5 does not exhibit these recognition features, but rather binds weakly to the internal regions of long duplex RNA (24,25). Therefore RIG-I and MDA5 are known to have preferences toward distinct RNA substrates: RIG-I recognizes short, duplex RNA with a 5′ triphosphate, whereas MDA5 binds long duplex RNAs in a cooperative manner (11,20,26–27). As implied by these molecular recognition features, RIG-I and MDA5 recognize different but overlapping groups of RNA viruses (8,28).

Several structures of RIG-I, MDA5 and MAVS have been reported which have greatly increased our understanding of the molecular basis of RLR signaling (7,29–30). Activation of RLR signaling is a carefully regulated process (4,7,13,31). The crystal structure of *apo* duck RIG-I suggests that the CARDs are trapped in an inactive conformation through intra-molecular interactions between CARD2 and HEL2i (32). Recognition of RNA alone may not be sufficient to activate RIG-I and thus adenosine triphosphate (ATP) binding has been suggested to play a role for RIG-I to exit from the auto-inhibited state by providing energy to fully release the CARDs (19,32–37). Post-translational modifications including ubiquitination, phosphorylation and non-covalent K63-linked polyubiquitin binding to the CARDs have been reported to be essential for full activation of RIG-I (36,38). More recently, two new structures—a human RIG-I CARDs tetramer bound to poly-ubiquitin chains and a RIG-I CARDs-MAVS CARD complex—were determined, providing the molecular basis for MAVS fiber formation (29,39–40). MDA5 binds long RNA duplex cooperatively and together they form helical fiber-like mega-structures through its RNA sensing module, the HEL-CTD (24,26–27,41–42). The CARDs of MDA5 connect to the HEL-CTD through a long, non-structured linker and it has been shown that the association of non-covalent K63-linked polyubiquitin is also required for MDA5 CARDs activation (33).

Hydrogen/deuterium exchange coupled with mass spectrometry (HDX-MS) has emerged as a sensitive way to study protein folding, protein-ligand interaction and protein intra- and inter-molecular interactions in solution, providing dynamic structural data (43–46). Backbone amide hydrogen exchanges with deuterium at a rate that is measurable by MS. Given solvent accessibility and structure stability, different regions on a protein or the same protein region in different states can become deuterated at different rates and to different extents. The differential hydrogen deuterium exchange events measured by MS are mapped to the primary sequence and structure models of the protein (47). For example, to study the mechanism of ligand activation of the vitamin D receptor, HDX-MS has been applied to map conformational dynamics of its ligand binding domains in complex with different chemical compounds (44). HDX-MS has also been used to show the subdomain structural movements of the viral packaging motor P4 upon RNA binding and ATP hydrolysis (46), and to characterize subunit conformational dynamics of the HIV-1 capsid protein during its maturation process (48).

Studies have pointed out that multiple states and sequential conformational changes are essential for the activation of mammalian RIG-I and MDA5 (24,26,33,41–42,49). Some of these states and conformations remain hypothetical and await stringent experimental validation. First, we chose biologically relevant RNA ligands—short 5′ triphosphorylated 10 base pair hairpin RNA and long duplex RNA polyIC—as RNA Pathogen-associated molecular patterns (PAMPs) to activate RIG-I and MDA5 and performed HDX-MS to visualize the activation process. Our data provide the first in-solution evidence that full-length human RIG-I adopts an auto-inhibitory conformation, whereas full-length MDA5 adopts a more extended conformation. Next, using our functional HDX experiment, we recorded the unique allosteric effects upon sequential addition of RNA PAMPs and ATP during the RIG-I CARDs activation process. Then, we showed that MDA5 oligomerizes on polyIC in solution by mapping the cooperative binding surfaces on the MDA5 HEL-CTD and present a high-resolution model of a cooperative MDA5–RNA complex. Our HDX-MS analyses, together with previous structural studies, enable us to depict a detailed step-by-step view of the early events during RLR activation.

## MATERIALS AND METHODS

### Protein sample preparations

The constructs were cloned into the pET-SUMO vector (Invitrogen) and transformed into Rosetta II (DE3) *Escherichia coli* cells (Novagen). As described previously, the proteins were expressed in Luria Broth (LB) media at an OD_600_ of 0.8 upon the addition of 0.5-mM isopropyl-β-D-thiogalactopyranoside (IPTG) and grown at 18°C overnight for 20 h (20,34). The cells were then lysed with a homogenizer (GEA), clarified by centrifugation and purified by batch binding with Ni-NTA beads (Qiagen). After collection and elution from Biorad polyprep columns, the RIG-I constructs were further purified on a HiTrap Heparin HP column (GE Healthcare) followed by a HiPrep 16/60 Superdex 200 column (GE Healthcare). Protein preparations were concentrated to 5–10 mg/ml with a 50k MW cutoff Amicon centrifugal concentrator (Millipore), and concentrations were determined spectrophotometrically using the extinction coefficients of *ϵ* = 99 700 M^−1^ cm^−1^ for the human RIG-I, 60 280 M^−1^ cm^−1^ for the RIG-I (ΔCARDs:1-229), 115 971 M^−1^ cm^−1^ for the mouse MDA5, 82 992 M^−1^ cm^−1^ for the MDA5(ΔCARDs:1-306) proteins. The storage buffer contained 25-mM Hepes, pH 7.4, 150-mM NaCl, 5% glycerol, 5-mM β-ME. The protein preparations were flash frozen with liquid nitrogen and stored at −80°C. The purity for each protein was >95% as verified on sodium dodecyl sulphate-polyacrylamide gel electrophoresis (SDS-PAGE).

### RNA work

As described previously (20), 5′ triphosphorylated RNA hairpin (3p8L: 5′pppGGCGCGGC*UUCG*GCCGCGCC, 3p10L: 5′pppGGACGUACGU*UUCG*ACGUACGUCC) was transcribed with T7 polymerase and purified on 20% denaturing polyacrylamide gels. Low molecular weight (LMW) polyIC (Invivogen) was dissolved in buffer containing 25-mM Hepes (pH 7.4), 150-mM NaCl, 5% glycerol, 5-mM β-ME to a final concentration of 10-mg/ml. Concentrations were determined spectrophotometrically.

### Hydrogen/deuterium exchange

The buffer for HDX on-exchange was the same composition except that H_2_O was replaced with D_2_O (99.99%) and glycerol was excluded. Specifically, we added 1-mM ATP into D_2_O buffer (ATP&D_2_O buffer) for ATP hydrolysis studies associated with all proteins. All samples with and without RNA ligands were incubated on ice for 1 h prior to HDX. Four microliters of 18-μM RIG-I or RIG-IΔCARDs in the presence or absence of RNA ligand (100-μM 3p10L or 5-mg/ml polyIC) were mixed with 16 μl D_2_O buffer (final D_2_O concentration was 80%) or H_2_O buffer for 0-s samples, and incubated at 4°C at various time intervals for on-exchange, e.g. 10, 30, 60, 300, 900 and 3600 s. To study ATP hydrolysis, 4 μl 18-μM of RIG-I or RIG-IΔCARDs with respective RNA ligands (100-μM 3p10L and 5-mg/ml polyIC) were incubated on ice for 1 h before mixing the RNA–protein complex with 16-μl ATP&D_2_O or ATP&H_2_O buffer for 0-s samples, and incubated at 4°C at the same time intervals. Similarly, 4 μl of 18-μM MDA5 or MDA5ΔCARDs (with or without 5-mg/ml polyIC) were mixed with 16-μl D_2_O buffer (final D_2_O concentration was 80%) or H_2_O buffer for 0-s samples, and incubated at 4°C at various time intervals, e.g. 10, 30, 60, 300, 900 and 3600 s. To study ATP hydrolysis, 4 μl of 18-μM MDA5 or MDA5ΔCARDs with 5-mg/ml polyIC were mixed with 16-μl ATP&D_2_O or ATP&H_2_O buffer for 0-s samples, and incubated at 4°C at the same time intervals. Ice-cold 20 μl quench solution consisting of 1-M guanidine hydrochloride and 1.5% (v/v) formic acid were added to each on-exchanged sample after specific time intervals prior to rapid freezing by liquid Nitrogen until HDX-MS analysis. For fully deuterated control samples, 4 μl of 18-μM RIG-I (±200-μM 3p10L) or MDA5 (±5-mg/ml polyIC) were incubated with 16-μl D_2_O solvent at 37°C for 20 h. The following steps were performed in the same manner as other HDX time point samples. Three replicates were performed for each HDX time point.

For RNA ligand-RIG-I activation studies, 4 μl of 18-μM RIG-I were incubated with 100-μM 3p8L or 100-μM 3p10L or 5-mg/ml polyIC on ice for 1 h prior to HDX experiment. Four microliters of each sample were then mixed with 16 μl of D_2_O buffer for 1 h, quenched and analyzed by liquid chromatography (LC)-MS. Ice-cold 20 μl quench solution consisting of 1-M guanidine hydrochloride and 1.5% (v/v) formic acid were added to each on-exchanged sample after specific time intervals prior to rapid freezing by liquid Nitrogen until HDX-MS analysis. For ATP analog studies, 4 μl of 18-μM RIG-I were incubated with 100-μM 3p10L on ice for 1 h. Stabilized RIG-I&3p10L complex was then incubated with or without ATP/ADP/ADP-AlF_x_ (final concentration 1 mM in RIG-I&3p10L complex) for another hour to reach an equilibrium conformation prior to the HDX experiment. Four microliters of RIG-I&3p10L&ATP/ADP/ADP-AlF_x_ (18-μM RIG-I, 100-μM 3p10L and 1-mM ATP/ADP/ADP-AlF_x_ in final concentration) were then incubated with 16 μl D_2_O buffer for 5 min, quenched and analyzed by LC-MS. Ice-cold 20 μl quench solution consisting of 1-M guanidine hydrochloride and 1.5% (v/v) formic acid were added to each on-exchanged sample after specific time intervals prior to rapid freezing by liquid Nitrogen until HDX-MS analysis. The following steps were performed in the same manner as other HDX time point samples. Three replicates were performed for each HDX time point.

### LCMS under quench condition

For capillary-flow LC, buffer A was H_2_O containing 0.3% (v/v) formic acid, and buffer B was acetonitrile containing 0.3% (v/v) formic acid. Protein samples were then digested online by passing through an immobilized pepsin-coupled column (2.1 mm i.d. × 30 mm) (Invitrogen) and were de-salted for 3 min on a house-packed C4 trap (0.75 mm i.d. × 10 mm, C4 beads purchased from Michrom). The mobile phase for online pepsin digestion was buffer A and the flow rate was 150 μl min^−1^ driven by the LC loading pump (Dionex 3000 RSLC). A 20-min gradient on a house-packed C4 column (0.3 mm i.d. × 50 mm, C4 beads purchased from Michrom) was used to separate and elute peptic peptides prior to MS analysis. All parts are connected by 1/16″ OD × 50-μM ID PEEK tubing and the flow rate was 15 μl min^−1^ driven by LC NC pumps (nano/capillary pumps) (Dionex 3000 RSLC). The gradient started from 5% buffer B and increased to 35% buffer B within 20 min, followed by washing with 90% buffer B for 3 min and equilibration with 1% buffer B for 5 min. MS raw files were acquired in the range of m/z 300–2000 (with mass resolution set to 100 000 at m/z 400) for 30 min in positive mode on an Linear Trap Quadropole (LTQ)-Orbitrap mass spectrometer (Thermo Fisher Scientific) equipped with an Electrospray ionisation (ESI) source (capillary temperature 275°C and spray voltage of 5 kV). All the HDX systems were strictly performed at 0°C (fully buried on ice and water) and online pepsin digestion was carried out at 16°C. Blank injections were made between every two samples to remove carryover peptides. Data for each time point were repeated three times. All HDX data were normalized to 100% D_2_O content, corrected for an estimated average deuterium recovery of 70% and analyzed by the software HDX Workbench (50). The HDX Workbench has built-in statistical software to evaluate the statistical significance using a one-way ANOVA and Tukey's multiple comparison tests. Initial peptic peptide identifications were performed with the same HDX setup as described above. Four microliters of protein sample (20 μM) were injected into the HDX-MS system. Product ion (MS/MS) spectra were acquired in linear ion trap LTQ with eight most abundant ions selected in the precursor (MS) scan with a 7.5-s exclusion time. MS and tandem MS files were extracted and searched by using Integrated Proteomic Pipeline (http://integratedproteomics.com/products/ip2/) for high-confident peptide identification (searching parameters: Precursor delta mass cutoff 20, Best peptide FP threshold 0.01, Best peptide delta mass threshold 10).

### Structure modeling

The full-length *apo* human RIG-I structure was modeled based on the *apo* duck RIG-I structure (PDB code: 4A2W) and the human RIG-I CTD structure (PDB code: 3LRR). The full-length *apo* mouse MDA5 structure was modeled based on the RIG-I CARDs (PDB code: 4NQK) and the human MDA5 HEL-CTD (PDB code: 4GL2). The homology modeling program is HHPred (http://toolkit.tuebingen.mpg.de/hhpred) (51).

## RESULTS

### Human RIG-I maintains an auto-inhibited conformation through intra-molecular CARD2-HEL2i interactions

**Figure 1.**
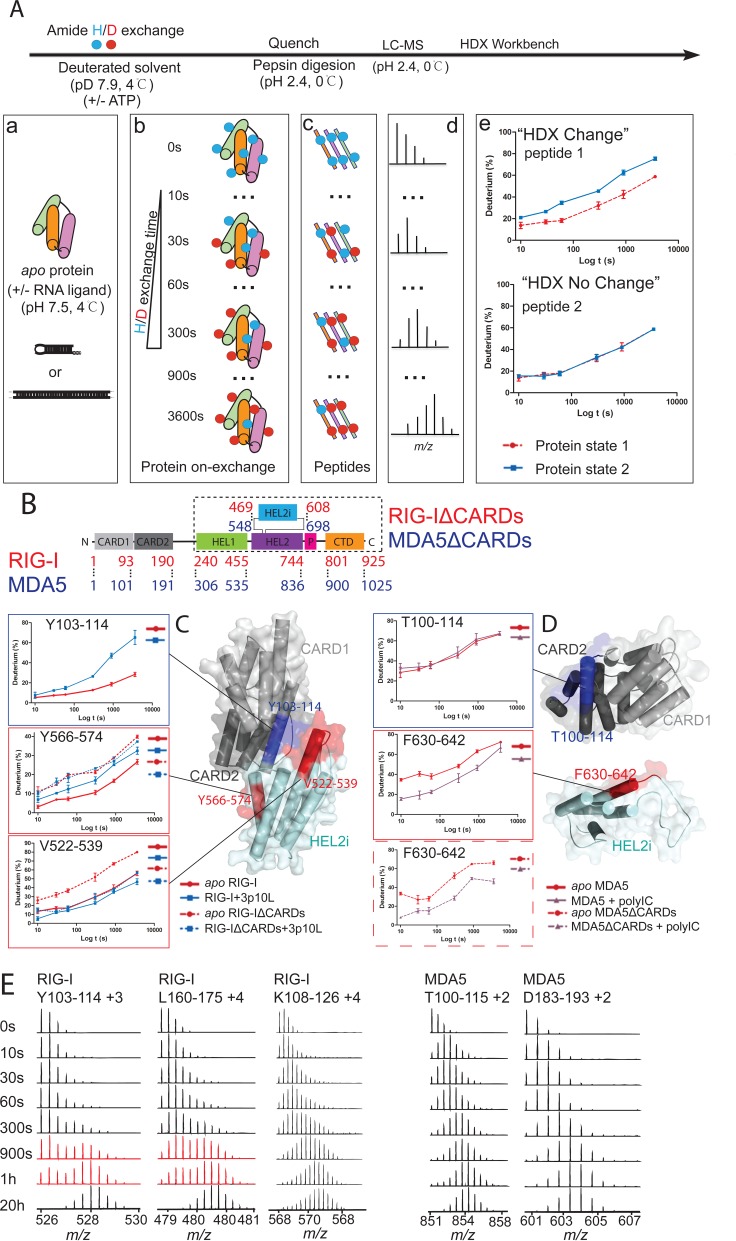
Apo states of RIG-I and MDA5. (**A**) Workflow of the differential HDX-MS experiments. (a) Differential HDX studies consisted of multiple experiments, e.g. *apo* protein, protein+RNA complex and protein+RNA&ATP. (b) Differential protein complexes were incubated with deuterated solvent at various time points under native conditions, followed by quenching (pH 2.4, 0°C) to terminate the HDX reactions. (c) Protein complexes were immediately digested by pepsin and highly reproducible peptides were separated by liquid chromatography under quenching conditions. (d) Mass spectrometry was then used to identify and characterize the deuterium-incorporated peptides. Differential HDX behavior was acquired in isotopic distribution for each peptide across various HDX time points. (e) The HDX Workbench provided the statistical analyses and graphical representations of the HDX-MS data (52). (**B**) Schematic representation of full length and truncated form of RIG-I and MDA5, displaying CTD (light orange), Pincer (light red), HEL1 (light green), HEL2 (light purple), HEL2i (light blue) and tandem CARDs (gray). RIG-IΔCARDs and MDA5ΔCARDs are illustrated in the dashed box. (**C**) The auto-inhibited conformation of *apo* RIG-I is maintained by the CARD2 and HEL2i domain (structure model based on duck RIG-I apo enzyme, PDB: 4a2w). Deuterium uptake plots of the CARD2 latch peptide (Y103-114) and the HEL2i gate peptides (Y566-574 and V522-539) from RIG-I and RIG-IΔCARDs are shown. The data are plotted as percent deuterium uptake versus time on a logarithmic scale. Red, blue plots represent the RIG-I (RIG-IΔCARDs) apoenzyme and the 3p10L bound state, respectively. (**D**) The CARD2-HEL2i interface is absent in the MDA5 apoenzyme (structure model based on the duck RIG-I CARDs, PDB: 4a2w and human MDA5ΔCARDs, PDB: 4gl2). Deuterium uptake plots of the CARD2 peptide (T100-114) and the HEL2i peptide (F630-642) from MDA5 and MDA5ΔCARDs are shown. The data are plotted as percent deuterium uptake versus time on a logarithmic scale. Red and purple plots represent the MDA5 (MDA5ΔCARDs) apoenzyme and + polyIC state, respectively. (**E**) The RIG-I CARD2 peptides Y103-114 and L160-175 follow EX1 kinetics (in red) with two distinct mass isotopic distributions. In contrast, the RIG-I CARD2 peptide Y108-128 and the MDA5 CARD2 peptides T100-115 and D183-193 follow EX2 kinetics (in black) in which only one mass isotopic distribution is present.

To examine whether mammalian RIG-I adopts an auto-inhibited conformation in solution similar to that observed in the static duck RIG-I structure, we performed HDX experiments with recombinant human RIG-I protein with and without ATP and RNA substrates. We also included truncated RIG-I (RIG-IΔCARDs, residues 229–925) as a control, which lacks the N-terminal CARDs and the linker region to the helicase domain (Figure 1B). We used the 5′ triphosphorylated 10-mer hairpin RNA (3p10L) as the RNA ligand, which has recently been shown to bind and activate RIG-I *in vitro* and in cell-based IFN production assays (20). As illustrated in Figure 1A, RLR proteins in different substrate-bound states were subjected to deuterated solvent over various time intervals followed by a quenching step in low pH on ice. All samples were then digested by low pH compatible pepsin enzyme. The peptides were subsequently separated by LC performed under quench conditions prior to being measured in a mass spectrometer, and the HDX Workbench was then utilized for statistical analysis and graphical representation(52). In total, 221 peptides from RIG-I and 194 peptides from RIG-IΔCARDs were identified and characterized across all HDX time points, representing a high sequence coverage of 89% for RIG-I and 86% for RIG-IΔCARDs (Supplementary Figure S1a and e).

In the *apo* state, the latch peptide Y103-114 on RIG-I CARD2 exhibited the greatest magnitude of protection to HDX with less than 30% deuterium uptake after a 1-h incubation in deuterated solvent. In contrast, in the presence of 3p10L, this protection was greatly compromised with deuterium incorporation of up to 70% during the same time interval (Figure 1C). Correspondingly, a 15% increase in average deuterium uptake was observed for this latch peptide at all HDX time points (Table 1). This RNA-dependent deuterium uptake was also clearly observed in three adjacent CARD2 peptides, such as K108-126 (9%), I139-155 (11%) and L160-185 (9%) (Table 1). These peptides reside next to the outermost α-helical latch peptide and face away from the RNA binding site within the HEL-CTD. The CARDs are known to be rigid α-helical bundles with highly stable secondary structural compositions (30,53). Therefore, the significant differences in deuterium uptake in CARD2 peptides suggest that CARD2 in *apo* RIG-I contains a relatively solvent-inaccessible surface patch, which becomes more solvent-exposed in the presence of RNA ligand. Importantly, the sequences of the aforementioned CARD2 peptides also lie at the interface between CARD2 and HEL2i in duck RIG-I (32).

**Table 1.**
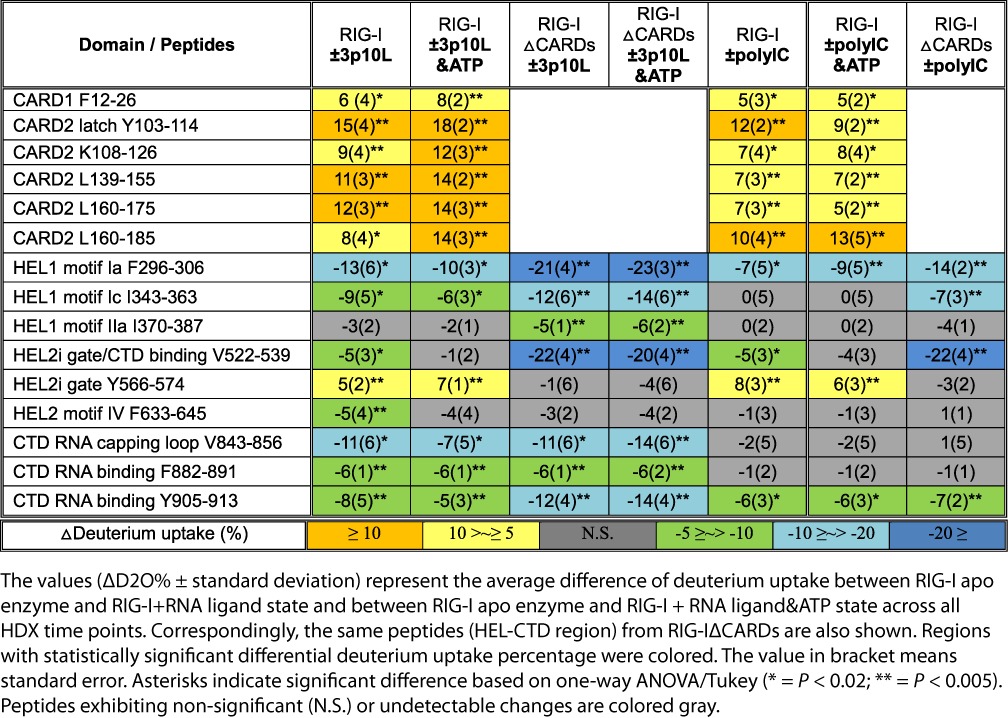
Comparison view of the conformational dynamics of RIG-I and RIG-IΔCARDs induced by RNA ligand (5′ triphosphorylated RNA hairpin 3p10L and polyIC) and ATP hydrolysis

RNA-induced deuterium uptake was also observed in the human HEL2i α-helical peptide Y566-574 in the full-length RIG-I protein. Specifically, a loss of protection (a 5% increase in average deuterium uptake at all HDX time points) for the gate motif Y566-574 was observed upon 3p10L RNA binding. In contrast, the same surface patch in the RIG-IΔCARDs showed statistically insignificant deuterium incorporations relative to the RNA bound state. Thus, RNA binding allosterically destabilizes the CARD2-HEL2i hydrophobic interface by releasing the RIG-I tandem CARDs. We also observed an intriguing HDX pattern in a second human HEL2i gate motif, V522-539. In *apo* RIG-I, peptide V522-539 exhibited an HDX kinetics pattern increasing from 15 to 55% over 1 h of HDX (Figure 1C). In contrast, peptide V522-539 from *apo* RIG-IΔCARDs exhibited a significantly higher deuterium uptake from 25 to 85% over the same time interval. Thus, the RIG-IΔCARDs V522-539 peptide displayed a higher degree of HDX in the absence of the tandem CARDs, further supporting that human *apo* RIG-I adopts an auto-inhibited conformation through intra-domain interactions between CARD2 and HEL2i. Interestingly, upon addition of 3p10L, we observed a 4-fold higher protection against HDX for RIG-IΔCARDs V522-539 (22%) compared to that of RIG-I (5%) (Table 1). We therefore reason that the V522-539 HEL2i peptide becomes protected from HDX by the CTD upon RNA binding following collaborative conformational rearrangements of the HEL and CTD domains. In the full-length RIG-I protein, however, the V522-539 peptide was protected from HDX in both the apo and RNA bound state. This is likely because the CTD displaces the CARDs upon RNA binding and maintains the protection of the V522-539 peptide in the RNA bound state. These data are supported by structural alignments of different RIG-I states which clearly indicate that the CTD would displace CARD2 in the RNA and ATP bound states (19).

Among the CARD2 peptides that exhibited an RNA-dependent increase in uptake of deuterium (Table 1), peptides Y103-114 and L160-175 displayed unique partial local unfolding events following bimodal EX1 HDX kinetics at the 15-min and 1-h time points (Figure 1E). In EX1 kinetics (also known as cooperative exchange/unfolding), the unfolding rate of the corresponding protein region is slower than the HDX rate. As a result, it gives rise to two distinct MS envelopes—the lower mass isotopic distribution represents molecules that have not unfolded/exchanged and the higher mass distribution depicts molecules that have undergone unfolding/exchange events. The majority of native proteins follow EX2 kinetics wherein only a single isotopic distribution increasing in mass is observed over time, oftentimes as a result of local structural fluctuations or perturbations (54). Although the EX1 signature normally exists only under protein denaturing conditions (detergent or extreme pH), its occurrence under physiological conditions can reveal important insights into the nature of protein structural dynamics in solution such as large-scale conformational changes (55–57). Specifically, peptide Y103-114 is the CARD2 latch peptide that directly controls the opening of the RIG-I CARDs signaling module, while peptide L160-175 is the C terminal α-helical peptide in CARD2 that is attached through a long 55-residue linker to the HEL domain. The EX1 kinetic data for these two peptides suggest that binding to RNA PAMPs induces large-scale conformational changes in RIG-I in which the tandem CARDs undergo partial unfolding at the region near the long linker (L160-175) triggering disengagement from HEL2i. These two regions are therefore critical in controlling the gated activation of the RIG-I CARDs.

In the absence of the RNA ligand, amide hydrogen within the RNA binding motifs of the HEL and CTD domains consistently exhibited greater exchange with the deuterium solvent (Table 1 and Supplementary Figure S1a). We therefore conclude that the human RIG-I helicase and CTD domains adopt an open and extended conformation in the absence of RNA. This is in perfect agreement with the structural observations of the unliganded RIG-I proteins (32,58–59).

### RIG-I binds and responds to different RNA species (3p8L, 3p10L and polyIC) differently

In order to confirm the opening of the CARD2-HEL2i interface as the first step of RIG-I activation and to validate the HDX-MS method to monitor the activation process, three RNA species—3p8L, 3p10L and polyIC—were used in parallel to stimulate RIG-I in our HDX-MS assay. 3p8L and 3p10L are short RNA hairpins with 5′ triphosphates but of different length (8 and 10 base pairs). 3p8L was shown to bind RIG-I but not elicit RIG-I-mediated interferon production in a cell-based reporter assay, whereas 3p10L both bound and effectively stimulated an RIG-I-mediated interferon response (19,20). In contrast to the short RNA hairpins, polyIC is mixture of long duplex RNAs bearing 5′-diphosphates and has been well known to activate RIG-I in a variety of assays (16,20,22). However, the molecular basis of how RIG-I reacts to these structurally different RNA species is still unknown.

**Figure 2.**
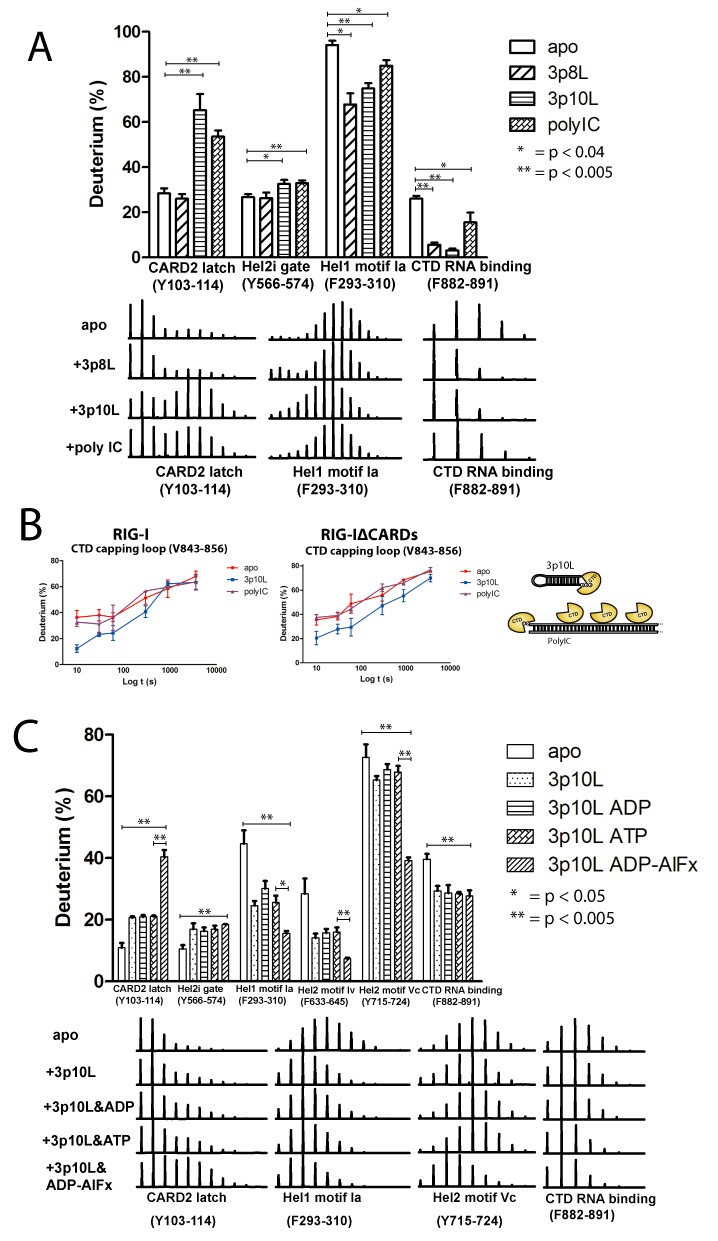
Characteristics of RNA and ATP binding to RIG-I. (**A**) Characteristic HDX profiles of RIG-I peptides induced by different RNA ligands. Deuterium uptake for peptides (Y103-114, Y566-574, F293-310 and F882-891) after a 1-h exposure to deuterated solvent. Asterisks indicate significant differences based on one-way ANOVA/Tukey between apo and +RNA states (* = *P* < 0.04; ** = *P* < 0.005). Isotopic distributions of selected peptides are shown for indicated states. (**B**) Deuterium uptake plots of RIG-I CTD capping loop. Deuterium uptake plots of peptide (V843-856) in RIG-I and RIG-IΔCARDs are shown. The data are plotted as percent deuterium uptake versus time on a logarithmic scale. Red, blue and purple plots represent RIG-I *apo* enzyme, +3p10L and +polyIC state, respectively. The right panel illustrates how the CTD binds 3p10L (end capping) and polyIC (end capping and internal binding) differently. (**C**) Characterization of ATP analog effects on RIG-I domain peptides. Deuterium uptake for peptides (Y103-114, Y566-574, F293-310, F633-645, Y715-724 and F882-891) in different ATP analog binding states after a 5-min exposure to deuterated solvent. Asterisks indicate significant differences based on one-way ANOVA/Tukey (* = *P* < 0.05; ** = *P* < 0.005). Isotopic distributions of selected peptides are shown for indicated states.

HDX profiles of polyIC bound RIG-I displayed significantly different characteristic patterns of RNA binding compared to that of 3p10L (Table 1). The most interesting HDX pattern was observed at the RIG-I CTD domain, in which the RNA capping loop V843-856 exhibited comparable HDX kinetics between apo and polyIC bound states, indicating this loop is not likely involved in binding to polyIC (Table 1 and Figure 2B). In contrast, the same peptide showed a high degree of protection against HDX (11% decrease in deuterium uptake) upon binding to the blunt-ended terminus of 3p10L (Table 1 and Figure 2B). Another RNA binding peptide F882-891 in the CTD also showed statistically insignificant changes in the HDX profiles upon polyIC binding (Table 1). The diphosphates at the 5′ ends of polyIC are likely to be the preferred binding sites of RIG-I especially during the earlier stages of the innate immune response where the physiological concentrations of RIG-I and RNA are low (16). However, in our HDX experiments, there are more internally bound RIG-I molecules than long polyIC duplexes, and therefore the HDX signals were dominated by RIG-I molecules that bind internally (Table 1). Overall, the RIG-I CTD bound to polyIC weakly through a smaller interface as only peptide Y905-913 displayed 8 and 6% decreased deuterium uptakes in both the +3p10L and +polyIC states. Similarly, the overall binding strength of the RIG-I HEL domain to polyIC was weaker compared to 3p10L. For example, upon polyIC binding, HEL1 motif Ia (F296-306) showed a 7% decrease in deuterium uptake and several other conserved RNA binding motifs—Ic, IIa and IV—displayed no significant differences in their HDX profiles (Table 1). In contrast, various degrees of deuterium uptake were observed in conserved RNA binding motifs of 3p10L bound RIG-I, including motif Ia (13%), Ic (9%) and IV (5%). Furthermore, unlike MDA5, we observed no evidence of cooperative binding of RIG-I on polyIC, as RIG-I lacked the inter-protein contacts observed in the MDA5 filament (Tables 1 and 2 and Supplementary Figures S1c and S2a). Finally, polyIC was also able to disrupt the RIG-I CARD2-HEL2i interface in our HDX experiment (Figure 2A and Table 1). At the 1-h HDX time point, the CARD2 latch peptide Y103-114 exhibited greatly reduced protection from HDX (70 and 55%) upon binding to 3p10L and polyIC compared to the RIG-I apoenzyme (30%). The corresponding HEL2i gate peptide Y566-574 also displayed an increase in deuterium uptake in the 3p10L and polyIC group compared to apo RIG-I (Table 1 and Figure 2A).

**Table 2.**
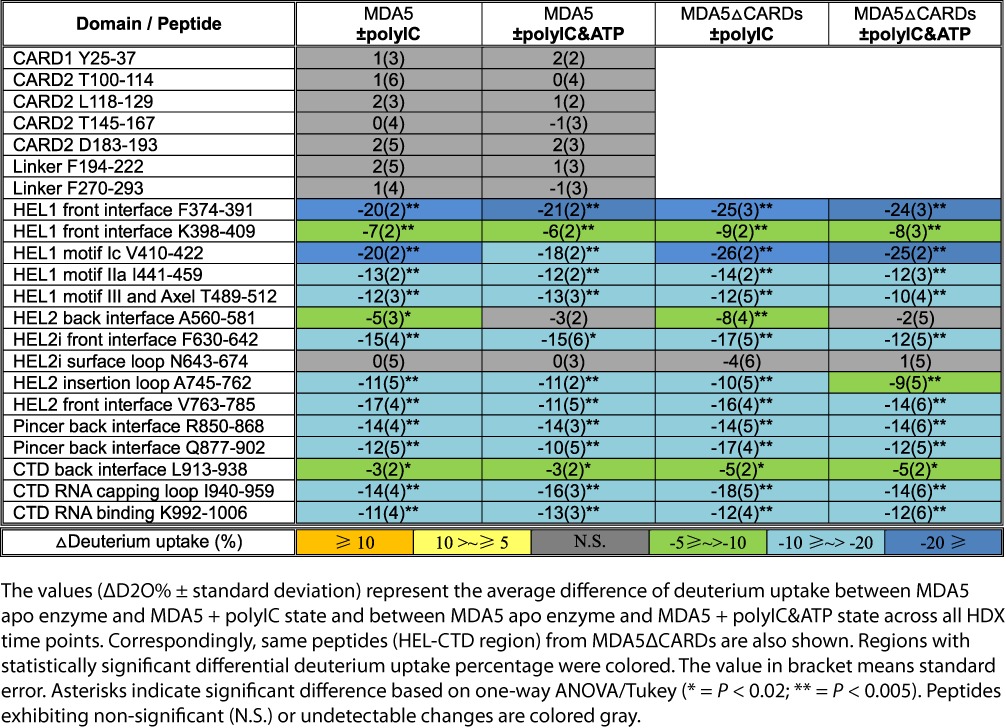
Comparison view of the conformational dynamics of MDA5 and MDA5ΔCARDs induced by polyIC and ATP hydrolysis

3p8L binds to the RIG-I HEL and CTD domains, as indicated by the HDX protection profiles of the HEL1 motif Ia F293-310 and CTD RNA binding peptide F882-891 (Figure 2A). However similar HDX rates in the CARD2 and HEL2i interface peptides were observed between *apo* RIG-I and 3p8L bound RIG-I, indicating that 3p8L is unable to allosterically disrupt the CARD2-HEL2i interface, thus explaining why 3p8L cannot elicit an RIG-I-mediated interferon response. This observation provides direct structure-based evidence in solution that the minimum length requirement of RNA PAMP is at least 10 base pairs.

### ATP allosterically modulates conformational rearrangements of RIG-I RNA complex

We next examined the role of ATP in driving the sequential conformational rearrangements of RIG-I subsequent to RNA recognition. HDX perturbations of 3p10L bound RIG-I were compared in the presence and absence of ATP (Table 1). RNA binding regions—motif Ia and Ic on HEL1, motif IV on HEL2—consistently exhibited a discernible ATP-dependent HDX kinetic profile, in which deuterium uptake plots in the presence of ATP fell between the *apo* and RNA bound states (Supplementary Figure S3). This ATP-dependent HDX enhancement also applied to the CTD domain, as shown by the kinetic profiles of the RNA capping loop (V843-856) and RNA binding residues (Y905-913) (Supplementary Figure S3). These results suggest that the RIG-I:RNA complex becomes less stable upon ATP binding and hydrolysis, which may drive the helicase and CTD to partially disengage from contacts with the RNA ligand and reach a conformational equilibrium in which thermo fluctuations occur more freely.

Intriguingly, ATP binding and hydrolysis also affected the HDX kinetics of the CARDs allosterically. When ATP was present in the samples of 3p10L bound RIG-I complex, RIG-I tandem CARDs consistently exhibited higher deuterium incorporations (Table 1). These ATP-induced effects extended through the CARDs landscape—with CARD2 peptide L160-185 demonstrating the biggest increase in deuterium uptake (from 8 to 14%) (Table 1). The CARD2 latch peptide Y103-114 exhibited an additional increase in deuterium uptake (from 15 to 18%) in the ATP-activated state (Table 1). Synchronous increase in HDX kinetics was also observed in CARD1 F12-26 (from 6 to 8%), suggesting that ATP binding and hydrolysis increase the structural dynamics of the CARDs allosterically (Table 1). It is also worth noting that CARD1 is known to retain a relatively constant conformation with respect to CARD2 (30,32). Furthermore, the ATP-enhanced HDX effects also took place on HEL2i gate motifs. Gate peptide Y566-574 exhibited a 5% increase in deuterium uptake upon RNA binding. ATP further enhanced the HDX rate of the gate peptide to a 7% increase in deuterium uptake (Table 1). The rate of amide hydrogen exchange with peripheral deuterium is also governed by local stability or fluctuations (60,61). Comparative HDX results suggest that RNA binding alone induces RIG-I to adopt a partially opened state, in which the CARD2-HEL2i intra-molecular interaction is loosened and RIG-I tandem CARDs form a conformational equilibrium where local fluctuation occurs. ATP binding and hydrolysis fully activate RIG-I tandem CARDs by completely dissociating them from HEL2i motif. Intriguingly, this is consistent with the observations that CARD2 latch peptide and its C-terminal peptide underwent partial unfolding events in solution upon RIG-I binding to RNA alone.

To further dissect the role of ATP on RIG-I activation, we examined the allosteric effects of ATP, ADP and ADP-AlF_x_ (non-hydrolyzable ATP analog) on RNA binding and CARDs opening mechanics of RIG-I (Figure 2C). 3p10L bound RIG-I was incubated with ATP, ADP and ADP-AlF_x_, respectively, for 1 h before a 5-min HDX in deuterated solvent. In CARD2, the deuterium uptake of latch peptide increased more than 2-folds in ADP-AlF_x_ group (∼44%), compared to 3p10L alone, 3p10L+ADP and 3p10L+ATP (∼21%) groups (Figure 2C). In HEL domain, enhanced protection against HDX were observed for the conserved RNA binding motifs upon binding to ADP-AlF_x_. HEL1 motif Ia, HEL2 motifs IV and Vc exhibited ∼40% decrease in deuterium uptakes in ADP-AlF_x_ group compared to 3p10L, 3p10L+ADP and 3p10L+ATP groups (Figure 2C). However, in CTD, peptide F882-891 showed similar deuterium uptake events for all the 3p10L bound RIG-I in the presence or absence of ADP, ATP and ADP-AlF_x_. These observations suggest that the helicase of RNA bound RIG-I adopts a much more compact conformation upon binding to ADP-AlF_x_ while the CTD RNA binding peptide is irresponsive to ATP analog binding. RIG-I CARD2 became more open and accessible to the solvent upon ADP-AlF_x_ binding to the HEL domain.

### CARDs of *apo* MDA5 are exposed in solution

To determine the MDA5 conformation in its resting state and to compare it with RIG-I, we performed HDX experiments with recombinant mouse MDA5 and MDA5ΔCARDs (residues 306–1025) in parallel (Figure 1B). To assess the conformational changes that lead to MDA5 activation, we used polyIC as the RNA ligand and performed the HDX kinetic experiments in the presence of ATP. In total, 182 and 120 peptides were recorded and mapped to full-length MDA5 and MDA5ΔCARDs, representing 82 and 84% sequence coverage, respectively (Supplementary Figure S2a and c). We observed no significant difference in the perturbation view of the tandem CARDs between *apo* and polyIC bound states (Figure 1D and Table 2). In particular, MDA5 CARD2 peptide T100-114, structurally corresponding to the RIG-I latch peptide Y103-114, exhibited high amide hydrogen exchange activities irrespective of polyIC binding, yielding 70% deuterium uptake after 1 h incubation in deuterated solvent (Figure 1D). Its adjacent CARD2 peptides (L118-129, T145-167 and D183-193) and linker peptides (F194-222 and D271-293) consistently showed statistically insignificant HDX patterns (Table 2). Furthermore, unlike RIG-I, there were no partial unfolding events involved in MDA5 CARD2 peptides T100-115 and D183-193 (corresponding to RIG-I Y103-114 and L160-175) (Figure 1E). Therefore these uniform HDX rates associated with MDA5 CARDs suggest that it remains solvent-exposed in the presence and absence of polyIC.

To further highlight the radical differences between *apo* MDA5 and *apo* RIG-I, we observed that the peptide F630-642 on MDA5 HEL2i surface, corresponding to the RIG-I HEL2i gate motif, showed considerably higher deuterium uptake (a 15% increase in average deuterium uptake across all HDX time points) in the absence of polyIC (Figure 1D and Table 2). Furthermore, HDX kinetics of this peptide in full-length MDA5 was indistinguishable from that in MDA5ΔCARDs, which also showed higher deuterium exchange activities (a 17% increase in average deuterium uptake across all HDX time points) in the *apo* state (Figure 2D and Table 2). These results together indicate that the surfaces of MDA5 CARDs as well as HEL2i are not involved in the intra-molecular auto-inhibitory interactions as with RIG-I.

### Cooperative binding of MDA5 to polyIC is mapped to the HEL-CTD domain surface

**Figure 3.**
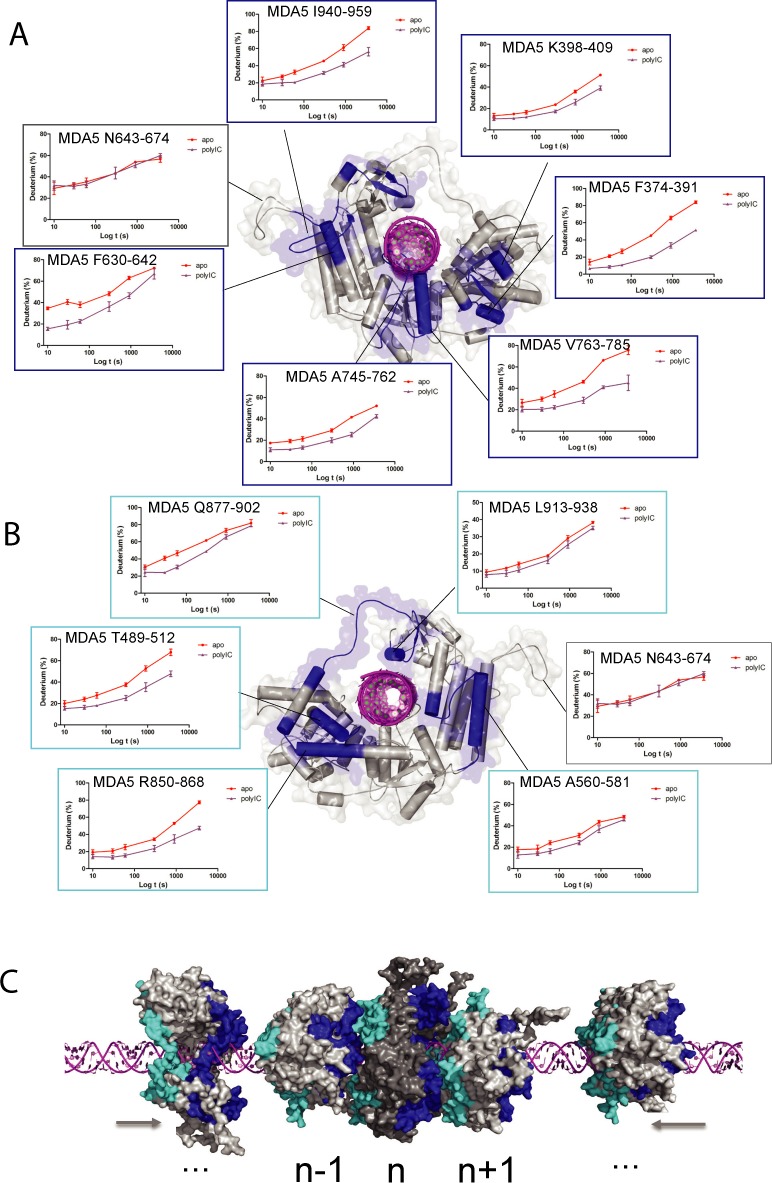
Structural basis of cooperative MDA5 binding to long duplex RNA. (**A**) The front view: deuterium uptake plots of MDA5 peptides involved in inter-molecular interactions (F374-391, K398-409, F630-642, A745-762, V763-785 and I940-959). The data are plotted as percentage deuterium uptake versus time on the logarithmic scale. Red plots indicate MDA5 *apo* enzyme and purple plots represent +polyIC state, showing that these surface peptides are more protected against HDX in the presence of polyIC. These peptides are colored in blue and mapped to MDA5 front surface. The HEL2i surface loop N643-674 (in gray), which is truncated in the reported crystal structure, is also modeled according to its HDX profile. (**B**) The back view: deuterium uptake plots of the peptides involved in inter-molecular interactions (T489-512, F630642, R850-868, Q877-902 and L913-938). (**C**) A model displays cooperative binding of three neighboring MDA5 monomers to the long dsRNA. This model is constructed based on our HDX data taking the previous cross-linking experiments into consideration (24,27,42). The front and back interface peptides of the central MDA5 monomer (n) are colored in blue and light blue according to (A) and (B). The interfaces of two neighboring MDA5 monomers (n−1 and n+1) are highlighted as well. See also the Supplementary Movie S1 on the cooperative binding of MDA5 to duplex RNA.

Since MDA5 forms cooperative filaments rather than monomeric complexes with RNA (22,26–27), obtaining high-resolution structural information of the functional unit of this protein remains challenging. To map out the molecular interface within the co-operative MDA5 polymer on a long duplex RNA, we recorded HDX kinetics for an MDA5:polyIC complex. MDA5 helicase and CTD formed extensive interactions with polyIC. In HEL1, residues V410-422, covering the helicase motif Ic, showed an over 20% decrease in deuterium uptake in the presence of polyIC (Table 2). Two HEL1 peptides, covering RNA binding motif IIa (I441-459), motif III and the Pincer axel (T489-514), also exhibited 13 and 12% less deuterium uptake, respectively. These data indicate HEL1 forms tight interactions with the RNA duplex in agreement with the structural observations (24). In HEL2, peptide A745-762 showed increased protection (11%) against HDX compared to the unliganded state. This peptide forms a loop inserting into the major groove of the duplex RNA (24). The CTD also established several direct interactions with polyIC. Peptide L913-938 showed a low extent of protection from deuterium exchange (a 3% decrease in HDX) upon polyIC binding, mainly because this peptide contains an α-helix and two β-sheets and is close to the RNA binding surface (24). Peptide K992-1006 on the CTD surface displayed a high degree of protection against HDX upon binding to polyIC, as shown by the marked 11% decrease in deuterium uptake. This peptide was observed to interact directly with one strand of duplex RNA (24). To further evaluate these RNA binding regions, we measured MDA5ΔCARDs HDX in response to polyIC binding. As expected, all above-mentioned RNA binding motifs in MDA5ΔCARDs showed similar degrees of protections from deuterium exchange to that of the full-length MDA5 (Table 2). Cooperative binding of MDA5 to dsRNA was clearly demonstrated by the polyIC-induced HDX resistant peptides that are not involved in RNA binding (Table 2). We identified 11 peptides mapped to the surfaces of HEL-CTD, which build up the ring-shaped RNA recognition module of MDA5. Six were clustered on one face of the ring (hereafter termed as the front face; Figure 3A) and five on the reverse face (back face; Figure 3B). These peptides formed the inter-molecular interface that mediated the cooperative binding of MDA5 to long RNA duplexes (26–27,42). On the front face, six peptides played important roles in establishing an inter-molecular interface between MDA5 monomers (Figure 3A and Table 2). In HEL1, peptide F374-391 showed significant protection against HDX with an apparent decrease of 20% deuterium uptake upon binding to polyIC. This peptide is closer to the outer rim of the MDA5 ring-shaped architecture. A second short α-helical peptide at the same region, K398-409, also recorded a drop of 7% in deuterium uptake in the presence of long polyIC. In HEL2, peptide V763-785 was observed with significantly increased protection (17%) against HDX in the presence of polyIC. This peptide occupies an α-helical framework in conjunction with the HEL2 insertion loop (24). In HEL2i, peptide F630-642 was identified as a HDX resistant peptide, which exhibited a 15% decrease in deuterium uptake upon binding to polyIC. This peptide occupies the C-terminus of one α-helix on the surface of HEL2i in the reported crystal structure, with intra-molecular interaction between HEL2i and the CTD stabilizing this region from deuterium exchange (24). In contrast, the adjacent residues N643-674, comprising an unstructured surface loop, displayed identical HDX kinetics between *apo* MDA5 and polyIC bound MDA5, indicating that this region is disordered and solvent-exposed even in the presence of RNA ligand (Figure 3A). The HDX kinetic profiles for the CTD peptide I940-959 are intriguing. In *apo* MDA5, over 80% deuterium uptake was observed after 1-h incubation in deuterated solvent, suggesting it was fully solvent-accessible in the absence of RNA. Upon polyIC binding, it only reached 60% deuterium uptake over the same time interval (Figure 3A). This CTD peptide is structurally equivalent to the RNA-capping loop in RIG-I, but is disordered in the crystal structure of the MDA5 dsRNA complex (24). Therefore, this protection against HDX is not likely due to RNA binding but rather inter-molecular interaction. Thus these six peptides from various subdomains of MDA5 provide an extensive contact surface for inter-molecular interactions. On the back face, five peptides were observed to form the other contact surface for the cooperative inter-molecular interaction (Figure 3B and Table 2). In HEL1, peptide T489-512 displayed increased protection against HDX (12%) upon polyIC binding. This peptide includes RNA binding motif III and the Pincer axel, and is also partially involved in RNA binding activities. Peptide A560-581 on the HEL2i surface also displayed increased protection against HDX (5%) in the presence of long dsRNA. Peptide R850-868 at the Pincer turn exhibited considerable protection against HDX (14%) in the presence of polyIC. Another peptide Q877-902 adjacent to the Pincer also showed a decrease of deuterium uptake (12%) compared to *apo* state. This peptide belongs to the loop connecting the HEL and CTD. Intriguingly, it is disordered in the crystal structure of MDA5–dsRNA complex (24), suggesting that this region might be more critical for inter-molecular interactions on long RNA duplex.

Previous cross-linking experiments have revealed that the helix α18 of HEL2 of one MDA5 monomer is in close proximity to the helix α10 on HEL2i of a second MDA5 molecule (24). Agreeable to that, peptide V763-785 (front face) is derived from the helix α18 and the helix α10 is partially overlapped with peptide A560-581 (back face). We propose a high-resolution model of the MDA5 inter-molecular interaction map that was refined based on these 11 HDX resistant peptides and previous cross-linking experiment. This model is in good agreement with the hypothesis that the front and back planes of MDA5 interface with each other at every ∼74° following the helical dsRNA backbone (Figure 3C and Supplementary Movie S1). In addition, these 11 peptides exhibited similar HDX kinetic profiles between full-length MDA5 and MDA5ΔCARDs (Table 2 and Supplementary Figures S3 and S4). These results unambiguously conformed that HEL and CTD domains of MDA5 concertedly mediate the cooperative recognition of long duplex RNA.

## DISCUSSION

### HDX-MS is an ideal tool to study the molecular mechanism of RLR signaling

Understanding the early activation process of the RLR signaling cascade promises to provide important insight into combating viral infection and consequently will be of great biomedical interest. However, the complete delineations of the RLR activation steps require high-resolution structures of full-length RIG-I and MDA5 captured in many different states. This is challenging if not impossible for the conventional methods such as crystallography and electron microscopy. To study the relatively large RLR *apo* enzymes, higher-order RNP complexes and the structural dynamics driven by ATP hydrolysis, we utilized HDX coupled with high-resolution MS to gain novel insights into the transitions of RIG-I and MDA5 from the resting state to the active state. In addition to full-length RIG-I and MDA5, we also designed parallel HDX studies on RIG-IΔCARDs and MDA5ΔCARDs. This experimental design allowed us to collect high coverage data for various conformational states (varying ligands and substrates). Indeed, we achieved high sequence coverage across seven HDX time points for RIG-I, MDA5 and the corresponding CARDs-deleted constructs. Direct comparisons of the time-resolved HDX-MS snapshots across these *apo*, RNA bound and ATP-stimulated enzymes enabled us to dissect the early activation steps of the RIG-I and MDA5 signaling cascades. Furthermore, our work also provided direct evidence for distinct RNA recognition and activation mechanics by using three RNA species of different lengths and secondary structure.

### The complete surveillance of virus RNA PAMPs is ensured by the different resting states of RIG-I and MDA5

**Figure 4.**
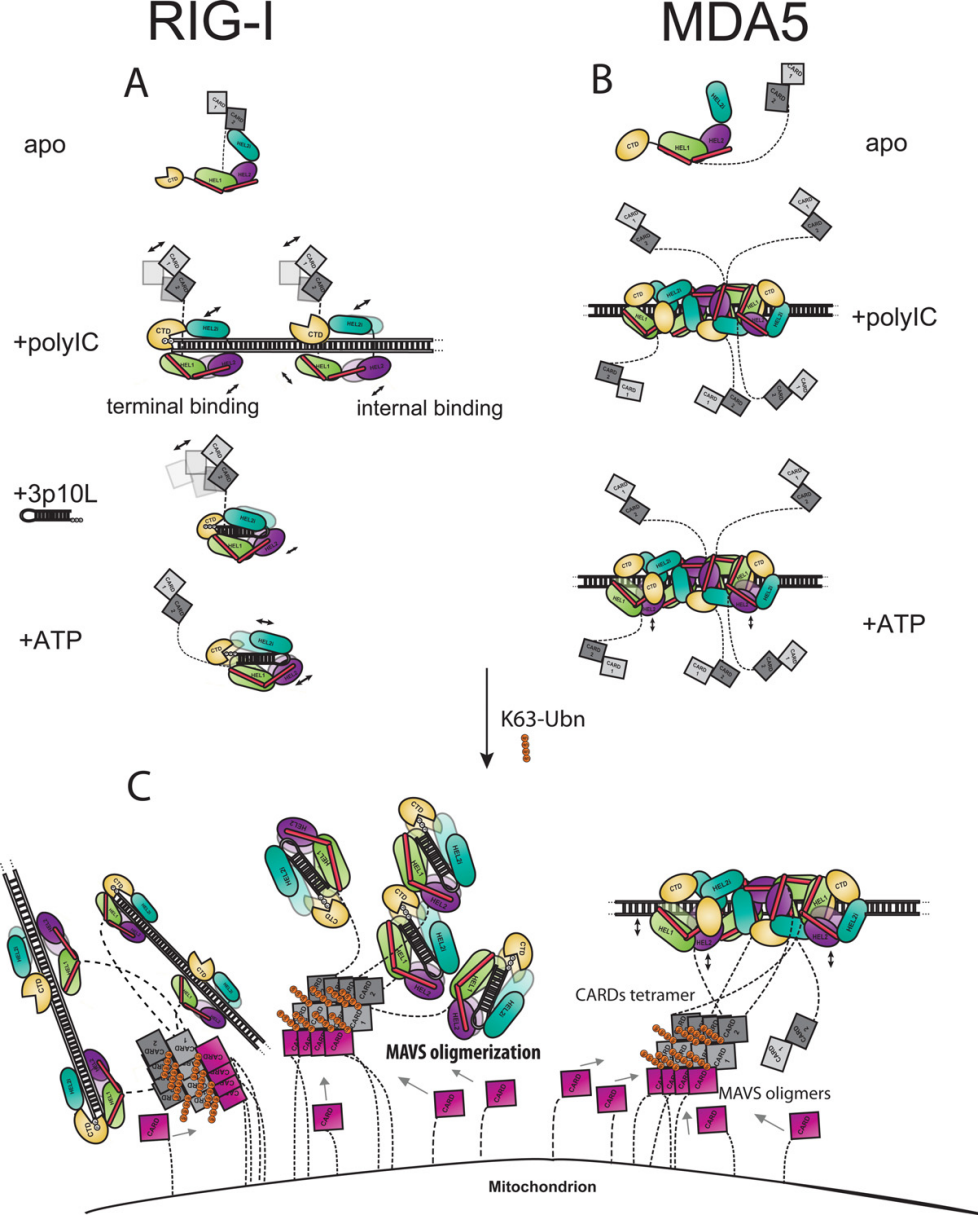
A model of the RIG-I and MDA5 activation pathways. (**A**) In the resting state, RIG-I adopts an auto-inhibited conformation via CARD2-HEL2i interaction while the HEL and CTD domains maintain a flexible and extended conformation available for sensing viral RNA. Binding of 3p10L triggers a drastic structural transformation resulted in a ring-shape architecture in which CTD captures the terminus of the duplex RNA and HEL clamps the backbone. RIG-I then forms a semi-opened conformation in which tandem CARDs are partially released from the gate motif on HEL2i. ATP binding results in complete disengagement of CARDs away from HEL2i (7,19). Upon binding to polyIC, RIG-I adopts a dynamic conformation in which both CTD and HEL bind to polyIC and CARDs dissociate from HEL2i. There is no cooperativity between individual RIG-I proteins. (**B**) MDA5 apoenzyme adopts an open and flexible conformation. Upon binding to polyIC, MDA5 proteins establish cooperative binding with neighboring molecules on the helical dsRNA backbone through their HEL-CTD rings. Five MDA5 molecules are estimated to form a repetitive unit (7,27,42). In this state, tandem CARDs are loosely connected to the HEL-CTD via a long linker. There is very little conformational change upon ATP binding and hydrolysis. (**C**) K63-poly ubiquitin chains assist RIG-I and MDA5 CARDs tetramerization on mitochondrial outer membrane and MAVS oligomerization then occurs via MAVS-CARD:RLR-CARDs interactions (6,30,33,36).

Our HDX data clearly show that human RIG-I adopts an auto-inhibited conformation in solution. This conformation is maintained by the intra-molecular interactions between the CARD2 latch peptide Y103-114 and the HEL2i gate motifs Y566-574 and V522-539. In this *apo* state, the HEL and CTD domains are flexible and fully solvent exposed. This model based on our HDX data agrees with the crystallographic studies on duck *apo* RIG-I, so it is likely that all vertebrate RIG-I proteins share a common auto-inhibited conformation in the resting state (Figure 4). In contrast, the HDX profiles of MDA5 reveal that MDA5 contains no intra-molecular contacts between the CARD2 and HEL2i domains as each domain is highly solvent-accessible in the *apo* state. In addition, MDA5 has a longer linker between the CARDs and HEL domains (∼100 disordered residues) compared to that of RIG-I (∼60 disordered residues). These intrinsic properties of RIG-I and MDA5 in their resting states ensure complete surveillance against virus infections by sensing all types of foreign RNAs which are heterogeneous both structurally and chemically (Figure 4).

### RIG-I and MDA5 are activated by separate and distinct mechanisms

Our HDX data indicate that for RIG-I, RNA loosens the CARD2-HEL2i intra-molecular interactions and then ATP binding further liberates the CARDs (Table 1 and Figure 4). We demonstrate that binding of 3p10L to RIG-I triggers a dramatic structural transformation in which both the HEL and CTD domains are bound tightly around the terminus of the duplex RNA. This RNP complex formation transforms RIG-I into a semi-open conformation in which the tandem CARDs are partially released and in conformational equilibrium with the gate motifs of HEL2i. We believe that ATP binding then alters the HEL-CTD-dsRNA architecture and further destabilizes the CARD2-HEL2i interaction (Table 1). We observe that ATP binding compresses the HEL-CTD-dsRNA complex, whereas ATP hydrolysis and ADP release result in disengagement of RNA from protein contact surfaces. Consequently, ATP drives the tandem CARDs completely away from HEL2i in a spatiotemporal manner as evidenced by the enhanced hydrogen bond exchange activities of the RIG-I tandem CARDs. Interestingly, following the addition of ATP we detected an increased deuterium uptake across RNA binding regions in the HEL and CTD domains that we attribute to thermo fluctuations from ATP-hydrolysis cycles. It is well established that the rate of amide hydrogen exchange with peripheral deuterium is governed by local stability or fluctuations (60,61).

Despite exhibiting no cooperativity upon binding to polyIC, the CARD2-HEL2i interface in RIG-I is clearly disrupted (Table 1 and Figure 4). As suggested by several structural studies of RIG-I, the RIG-I CTD primarily interacts with the alpha and beta 5′ phosphate of dsRNA (7,17–19,32,34–35,62). This was recently confirmed in an elegant functional study by Goubau *et al.* demonstrating that RIG-I can recognize the diphosphates at the 5′ ends of polyIC and trigger interferon production (16). Because polyIC are very long duplex RNAs with blunt, 5′ or 3′ heterogeneous ends, there are likely more internal-binding of RIG-I molecules than end-binding to long duplexes especially at high ratios of RIG-I to polyIC strands. Indeed, in our HDX experiments, HDX signals from RIG-I:polyIC and RIG-IΔCARDs samples were dominated by RIG-I molecules that bind internally (Table 1). Based on our data, it is evident that RIG-I possesses inherent flexibility in recognizing distinct RNA structures and in activating CARDs (Figure 2A and Table 1), although it is also clear that the overall binding affinity of the RIG-I HEL-CTD domains to polyIC internally is weaker compared to 3p10L with a triphosphorylated blunt end. The CTD plays the key role in modulating the two states of RIG-I (Figure 2B). Although it is not apparent under which states RIG-I is more potently activated, the presence of multiple binding long duplex RNAs preferably near the termini of polyIC or viral genomic RNAs will increase the local concentration of activated RIG-I and may trigger a more potent signaling event (16,20,23).

In contrast, we found that the MDA5 tandem CARDs are not locked into an auto-inhibitory state and show no remarkable changes in conformation in the presence or absence of polyIC or ATP (Table 2 and Figure 4). From our HDX experiments, we confirmed that the HEL-CTD of both the full-length and truncated MDA5 form a higher order complex with polyIC in agreement with previous structural studies (24). We identified eleven peptides on the non-RNA-binding surface of the HEL-CTD that were resistant to HDX. These peptides—six from the front face and five from the back face—formed large buried surfaces on both sides of the HEL-CTD ring-shaped architecture to mediate the cooperative binding of MDA5 (Figure 3). Introduction of ATP induced little or no changes to the MDA5-polyIC complex as no disruption of the cooperative interfaces was observed in either full-length MDA5 or MDA5ΔCARDs (Table 2). Our data explain why RIG-I and MDA5 play non-redundant roles in sensing pathogenic RNA species; these proteins require different activation signals, which could be a combination of the following factors: chemical and structural features of the RNA substrates, active concentrations of the RNA substrates and RLRs and the subcellular localizations of the RLR machinery (7,11).

In summary, our data illustrate the early events in the RLR activation pathways, which occur in a carefully choreographed order. RIG-I and MDA5 undergo completely different molecular pathways of activation (Figure 4), despite sharing high sequence similarities and conserved domain arrangements. RIG-I and MDA5 exist in different resting states, however both bind preferentially to distinct RNA ligands through their conserved HEL-CTD modules. For RIG-I, RNA binding occurs preferentially at the ends of dsRNA and partially releases its autoinhibitory closed conformation; ATP then further liberates the CARDs signaling module. Alternatively, RIG-I is also able to bind internally and becomes activated by long duplex RNAs. The structural flexibility of RIG-I enables the protein to recognize different RNA species distinctively and to activate its CARDs selectively, providing a molecular basis for the breadth of surveillance during infection by various viruses (11–12,63). In contrast, MDA5 exists in a relatively open CARDs conformation with a lower threshold of activation than RIG-I. Perhaps to compensate for this, MDA5 binds the stem of long dsRNA in a cooperative manner to form filamentous structures that place the CARDs physically close to each other. Taken together, we believe these different steps in the early activation of RIG-I and MDA5 serve the same purpose, which is to place the CARDs in a conformation and spatial arrangement to bind to their downstream adaptor protein MAVS and further activate the RLR signaling pathway by promoting the fiber-like oligomerization of MAVS (6,29,37).

## SUPPLEMENTARY DATA

Supplementary Data are available at NAR Online.

SUPPLEMENTARY DATA
