# Supplementary material for: High-resolution HDX-MS reveals distinct mechanisms of RNA recognition and activation by RIG-I and MDA5
Source: Nucleic Acids Res. 2014 Dec 24;43(2):1216–30. doi: 10.1093/nar/gku1329 (PMC4333383; doi:10.1093/nar/gku1329)
Supplement: SUPPLEMENTARY DATA [file supp_43_2_1216__index.html]

High-resolution HDX-MS reveals distinct mechanisms of RNA recognition and activation by RIG-I and MDA5 — SUPPLEMENTARY DATA 

# High-resolution HDX-MS reveals distinct mechanisms of RNA recognition and activation by RIG-I and MDA5

## SUPPLEMENTARY DATA

**Files in this Data Supplement:**

- SUPPLEMENTARY DATA
- SUPPLEMENTARY DATA
