# Supplementary material for: High-resolution HDX-MS reveals distinct mechanisms of RNA recognition and activation by RIG-I and MDA5
Source: Nucleic Acids Res. 2014 Dec 24;43(2):1216–30. doi: 10.1093/nar/gku1329 (PMC4333383; doi:10.1093/nar/gku1329)
Supplement: SUPPLEMENTARY DATA [file supp_gku1329_nar-03293-h-2014-File006.docx]

##### **Supplementary Information**

**High resolution HDX-MS reveals distinct mechanisms of RNA recognition and activation by RIG-I and MDA5**

Jie Zheng ^1^, Hui Yee Yong ^2^, Nantika Panutdaporn ^1^, Chuanfa Liu ^1^, Kai Tang ^1 *^, Dahai Luo ^2 *^

^1^ School of Biological Sciences, Nanyang Technological University.

60 Nanyang Drive, Singapore 637551.

^2^ Lee Kong Chian School of Medicine, Nanyang Technological University.

61 Biopolis Drive, Proteos Building, #07-03, Singapore 138673.

*To whom correspondence should be addressed.

Dahai Luo, [luodahai@ntu.edu.sg](mailto:luodahai@ntu.edu.sg) & Kai Tang, [ktang2004@gmail.com](mailto:ktang@pmail.ntu.edu.sg)

Present Address: Kai Tang, Silicon Kinetics Inc., 10455 Pacific Center Court, San Diego, CA 92121, USA

**Inventory of Supplemental Information**

**Supplementary Figure S1. Related to Table 1**

**Supplementary Figure S2. Related to Table 2.**

**Supplementary Figure S3. Deuterium uptake curves of selected peptides of RIG-I.**

**Supplementary Movie 1. Inter-molecular cooperativity of MDA5**

**Supplementary references**

**Supplementary Figure S1**

Differential hydrogen/deuterium exchange data for RIG-I is summarized. Differential deuterium exchange data for RIG-I in the presence and absence of (a) 3p10L and (b) 3p10L&ATP and (c) polyIC and (d) polyIC&ATP are shown using a perturbation view by HDX Workbench (Pascal, bruce). Correspondingly, differential deuterium exchange data for RIG-IΔCARDs in the presence and absence of (e) 3p10L and (f) 3p10L&ATP and (g) polyIC are shown using a perturbation view by HDX Workbench. Peptides are presented using rectangular strips below the respective portion of the protein sequence. Colors are used to characterize difference in average deuterium uptake for each peptide. The color key shows the colors assigned to deuterium differences (ΔD%) and the grey color represents no significant change. In RIG-IΔCARDs, conserved RNA binding motifs in the helicase domain consistently showed strong protection against HDX. This RNA binding effect is more obvious in RIG-IΔCARDs than RIG-I as the differences in deuterium uptake are larger for all RNA binding motifs in HEL (Table 1a). For instance in RIG-IΔCARDs, peptide F296-310 on HEL1 motif Ia exhibited an increased protection (21 % and 14%) against HDX in the presence of 3p10L and polyIC whereas this peptide in RIG-I showed almost half the extent of protection (13 % and 7%) upon binding to the same RNA ligand. Tighter interactions to polyIC were also shown in RIG-IΔCARDs compared to RIG-I that motif Ic exhibited enhanced HDX protection (7%) in the truncated RIG-I. These results are consistent with the fact that RIG-IΔCARDs has higher RNA binding affinity than RIG-I ([Vela et al, 2012](#_ENREF_7)). In contrast, CTD peptides showed similar HDX profiles between RIG-I and RIG-IΔCARDs in response to the same RNA ligand (3p10L or polyIC). The CTD capping loop in both full length and truncated RIG-I showed 11% decreases in deuterium uptake and statistical insignificance upon 3p10L and polyIC binding, respectively. Furthermore, ATP binding and hydrolysis exhibited distinct HDX profiles between 3p10L bound RIG-I and RIG-I△CARDs. Upon introduction of ATP in the presence of RNA, slightly increased protections against HDX were observed across RNA binding motifs in all subunits, including HEL1, HEL2, HEL2i, and CTD (Table 1). These results are consistent with the crystallographic observations that ATP bound RIG-I△CARDs forms a more compact conformation by interacting with both strands of dsRNA ([31](#_ENREF_31),[34](#_ENREF_34)). In the complex without ATP, RIG-I△CARDs is less compact as the helicase domain adopts a relatively open state wherein HEL2 is disordered ([33](#_ENREF_33)).

**Figure S1a: Differential HDX data for RIG-I ± 3p10L**


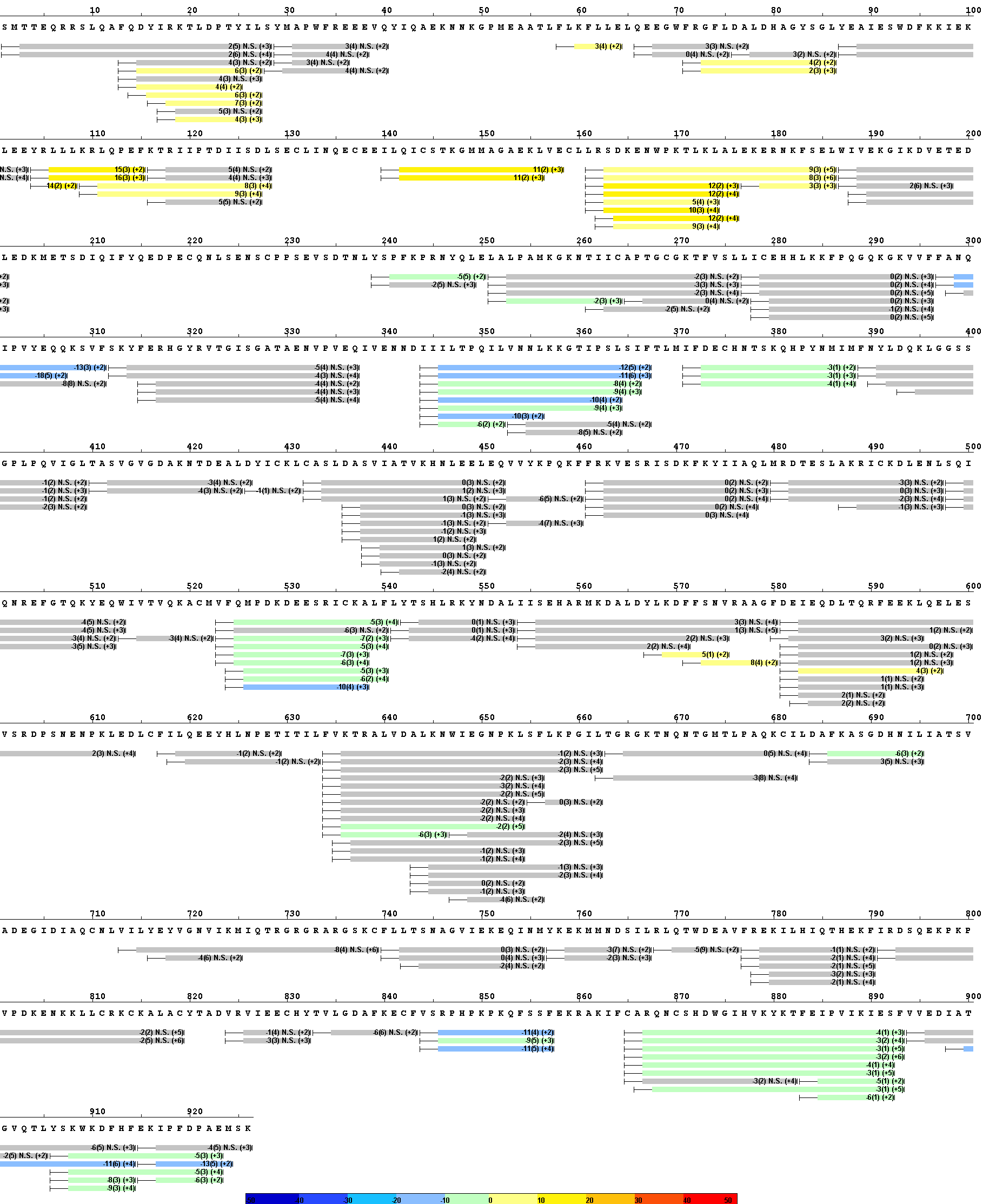


**Figure S1b: Differential HDX data for RIG-I ± 3p10L&ATP**

**
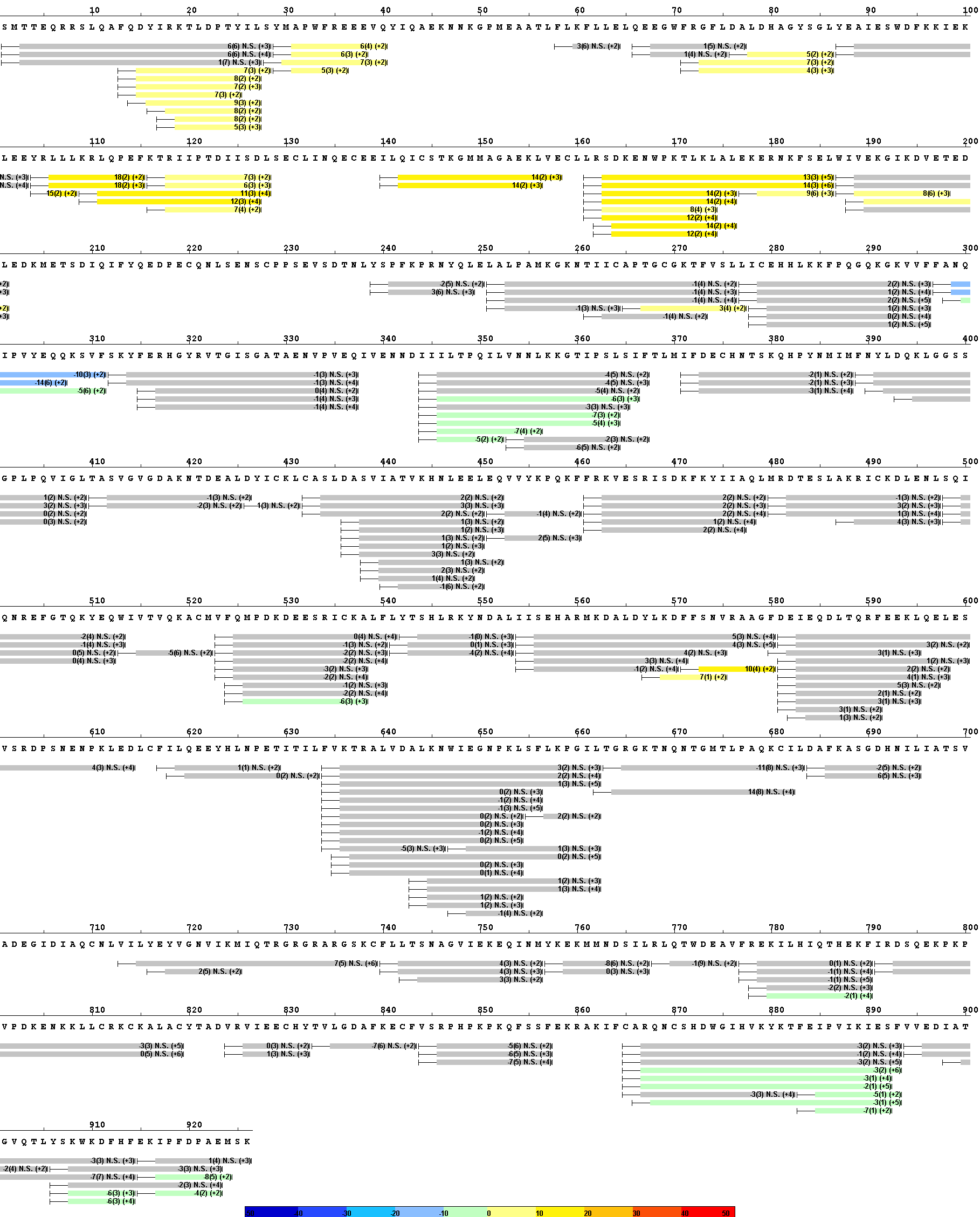
**

**Figure S1c: Differential HDX data for RIG-I ± polyIC**

**
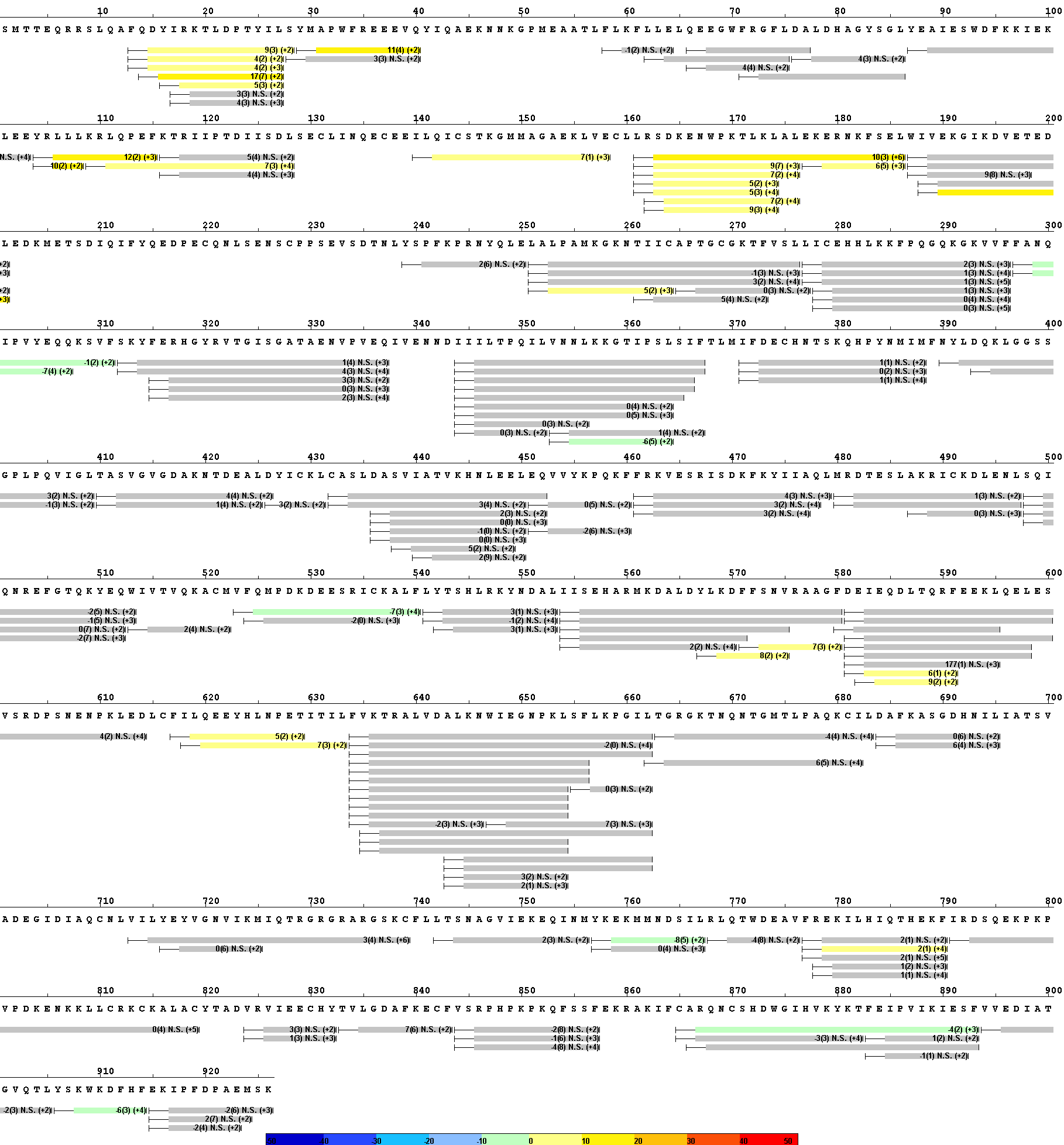
**

**Figure S1d: Differential HDX data for RIG-I ± polyIC&ATP**

**
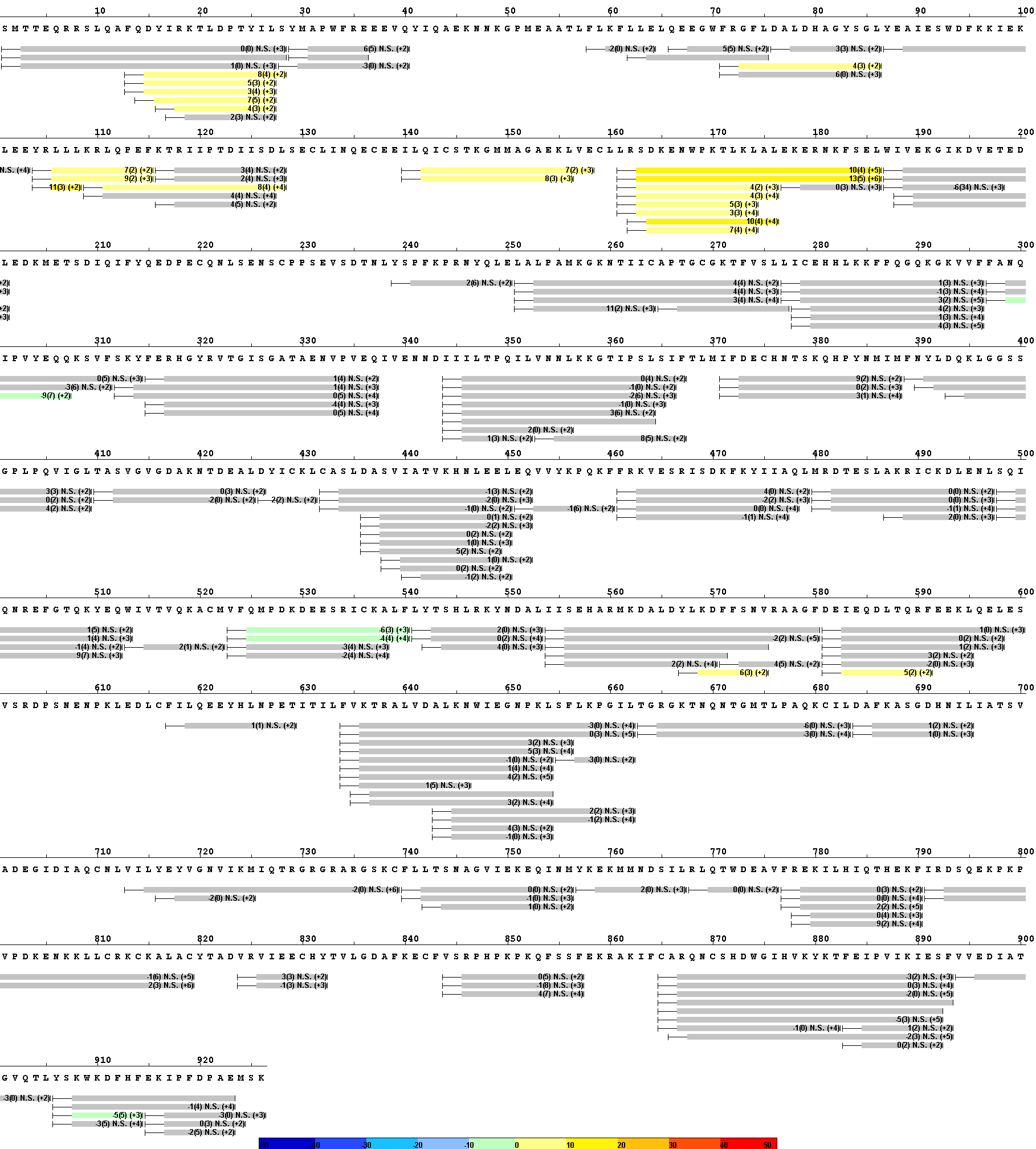
**

**Figure S1e: Differential HDX data for RIG-IΔCARDs ± 3p10L**

**
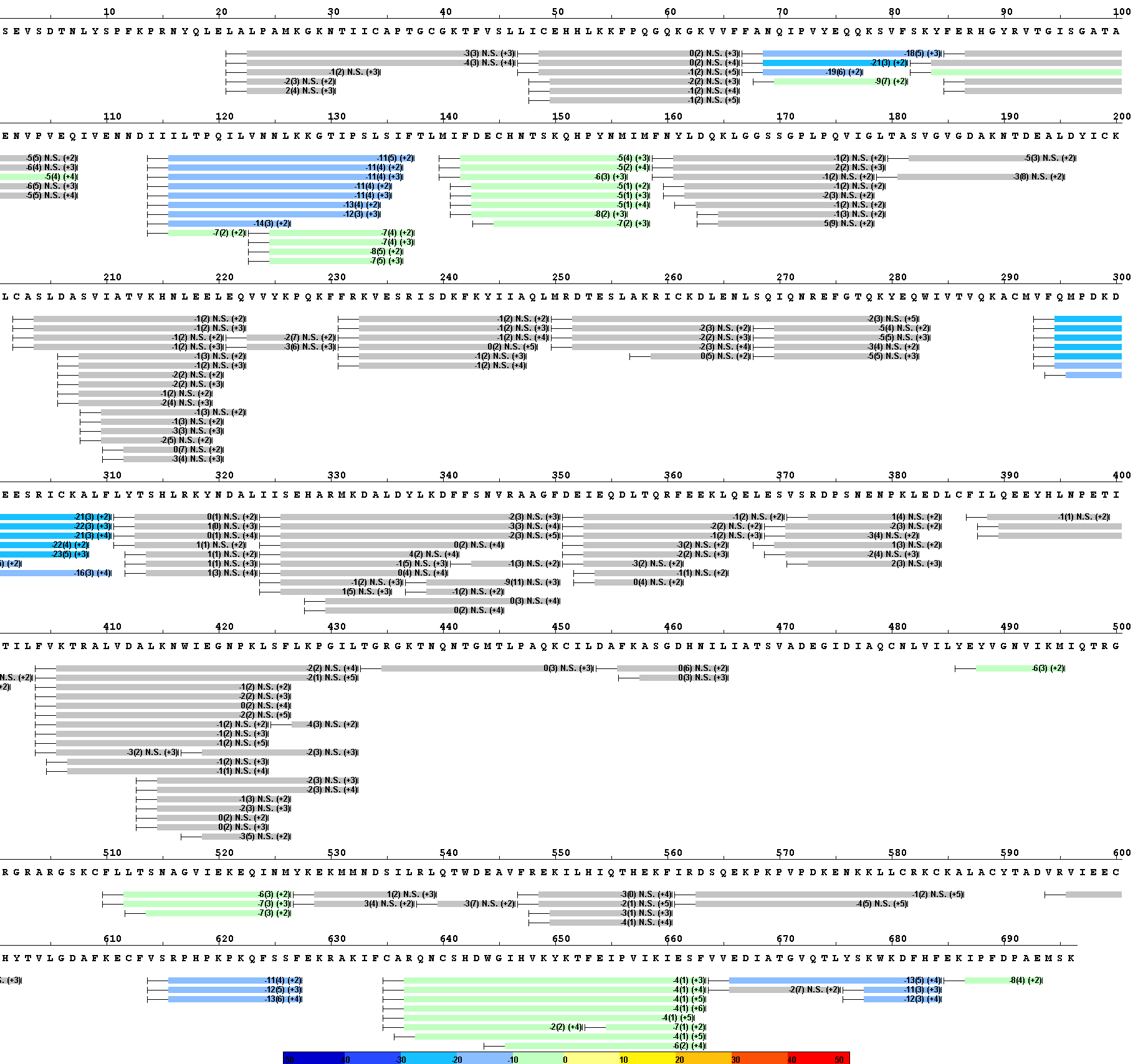
**

**Figure S1f: Differential HDX data for RIG-IΔCARDs ± 3p10L&ATP**

**
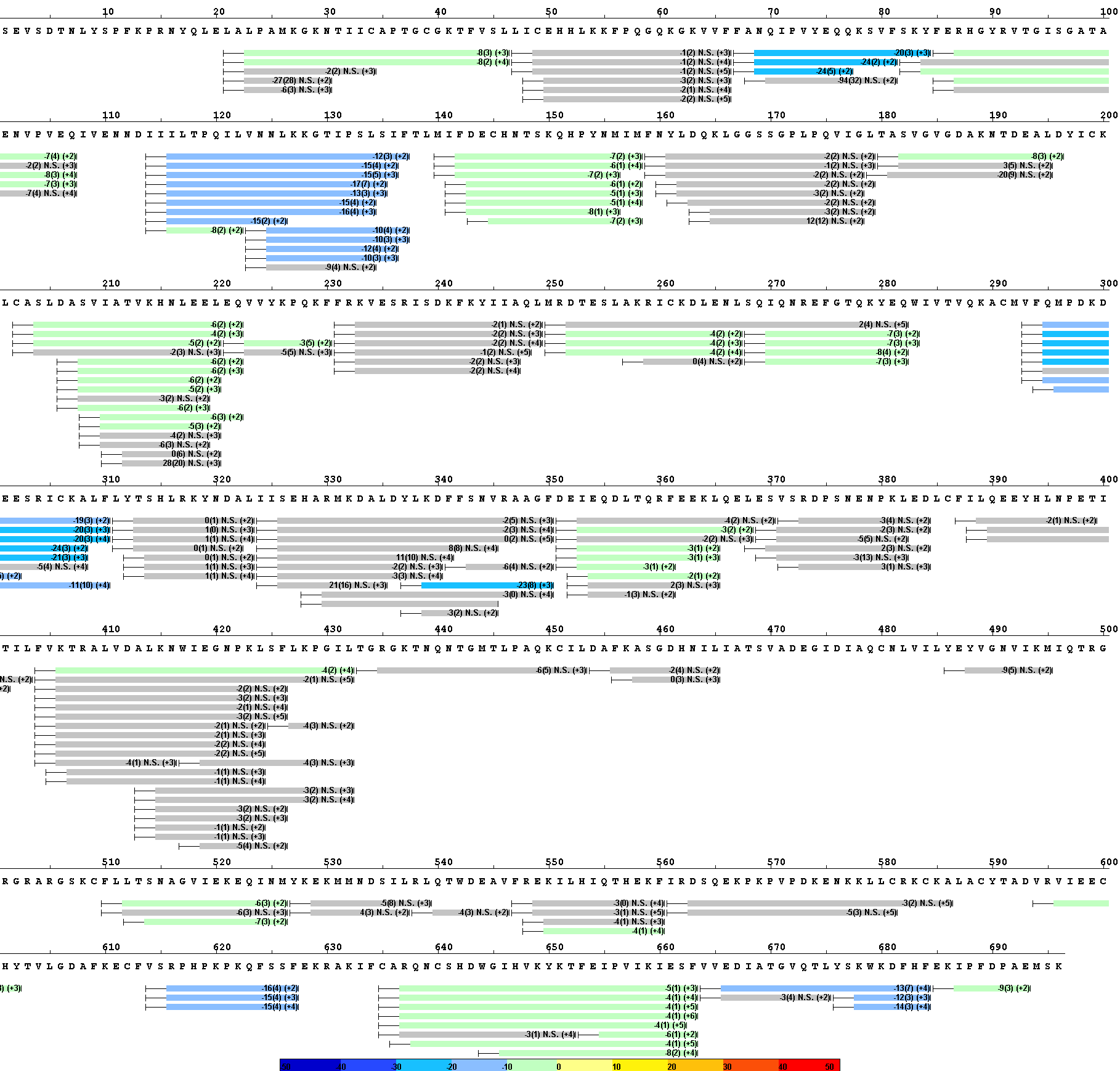
**

**Figure S1g: Differential HDX data for RIG-IΔCARDs ± polyIC**

**
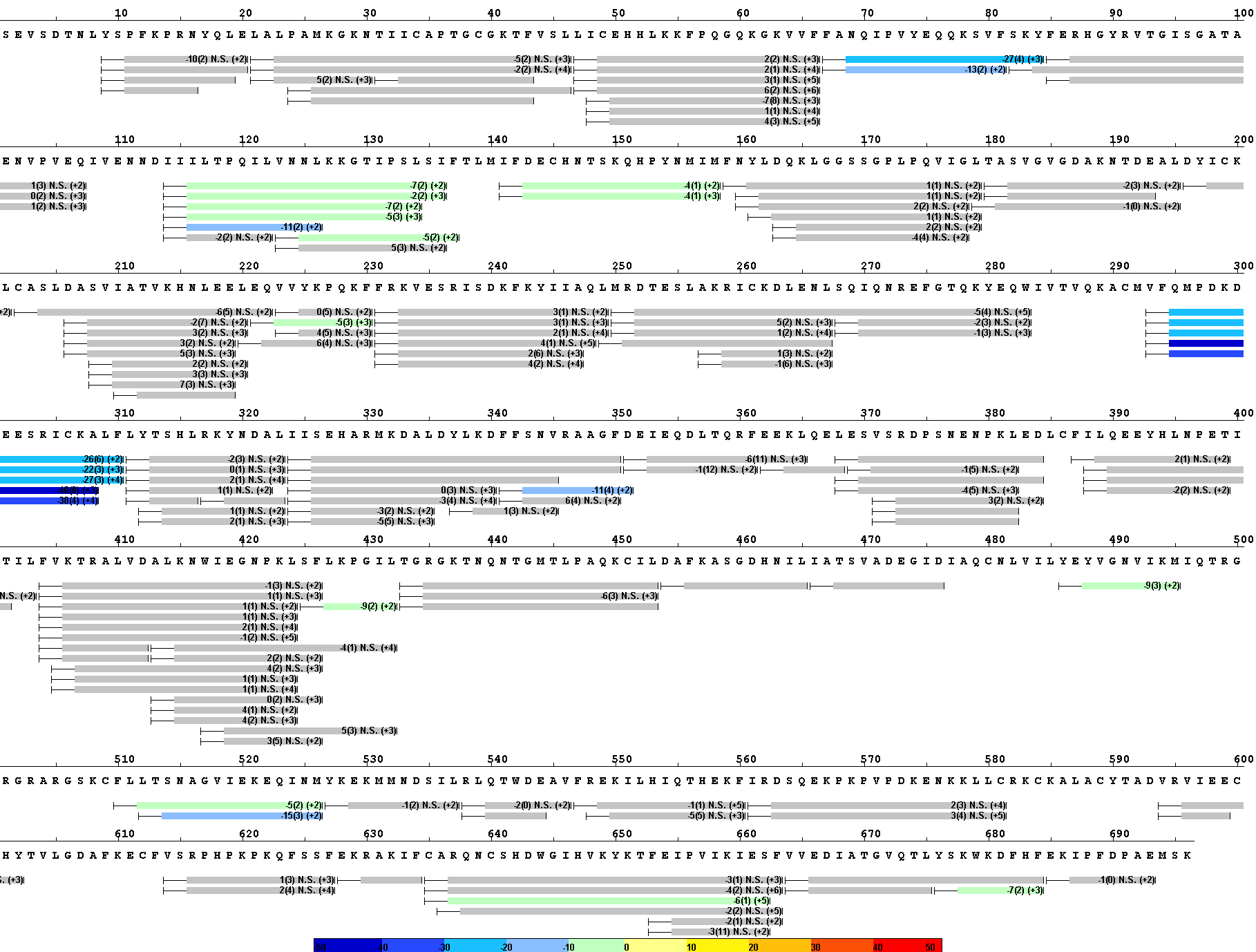
**

**Supplementary Figure S2**

Differential hydrogen/deuterium exchange data for MDA5 is summarized. Differential deuterium exchange data for MDA5 in the presence and absence of (a) polyIC and (b) polyIC&ATP are shown using a perturbation view by HDX Workbench (Pascal, bruce). Correspondingly, differential deuterium exchange data for MDA5ΔCARDs in the presence and absence of (c) polyIC and (d) polyIC&ATP are shown using a perturbation view by HDX Workbench. Peptides are presented using rectangular strips below the respective portion of the protein sequence. Colors are used to characterize difference in average deuterium uptake for each peptide. The color key shows the colors assigned to deuterium differences (ΔD%) and the grey color represents no significant change. Comparing Figure S2b&d with Figure S2a&c, ATP slightly weakens the interactions between MDA5 and polyIC but does not disrupt cooperativity. The interactions between MDA5 HEL1 domain and polyIC were also weakened in the presence of ATP (Table 2). RNA binding peptides from HEL1 motif Ic and IIa exhibited slight increases in deuterium uptake upon ATP binding and hydrolysis. The HEL1 motif Ic V410-422 demonstrated less than 2 % increase in deuterium uptake in the presence of ATP (from -20% to -18%). In addition, this ATP dependent disruption of RNA binding was also not prominent in MDA5ΔCARDs (Table 2). Of note, the eleven peptides that were identified to be involved in intermolecular cooperative binding had almost no ATP dependent HDX perturbation. Unlike RIG-I CARDs, ATP binding and hydrolysis did not alter the HDX kinetics of the CARDs of MDA5 (Table 2). We conclude that ATP only weakens the interactions between MDA5 and RNA but not the intermolecular interactions of MDA5, and that the CARDs of MDA5 is unaffected by ATP binding and hydrolysis (Table 2).

**Figure S2a: Differential HDX data for MDA5 ± polyIC**

**
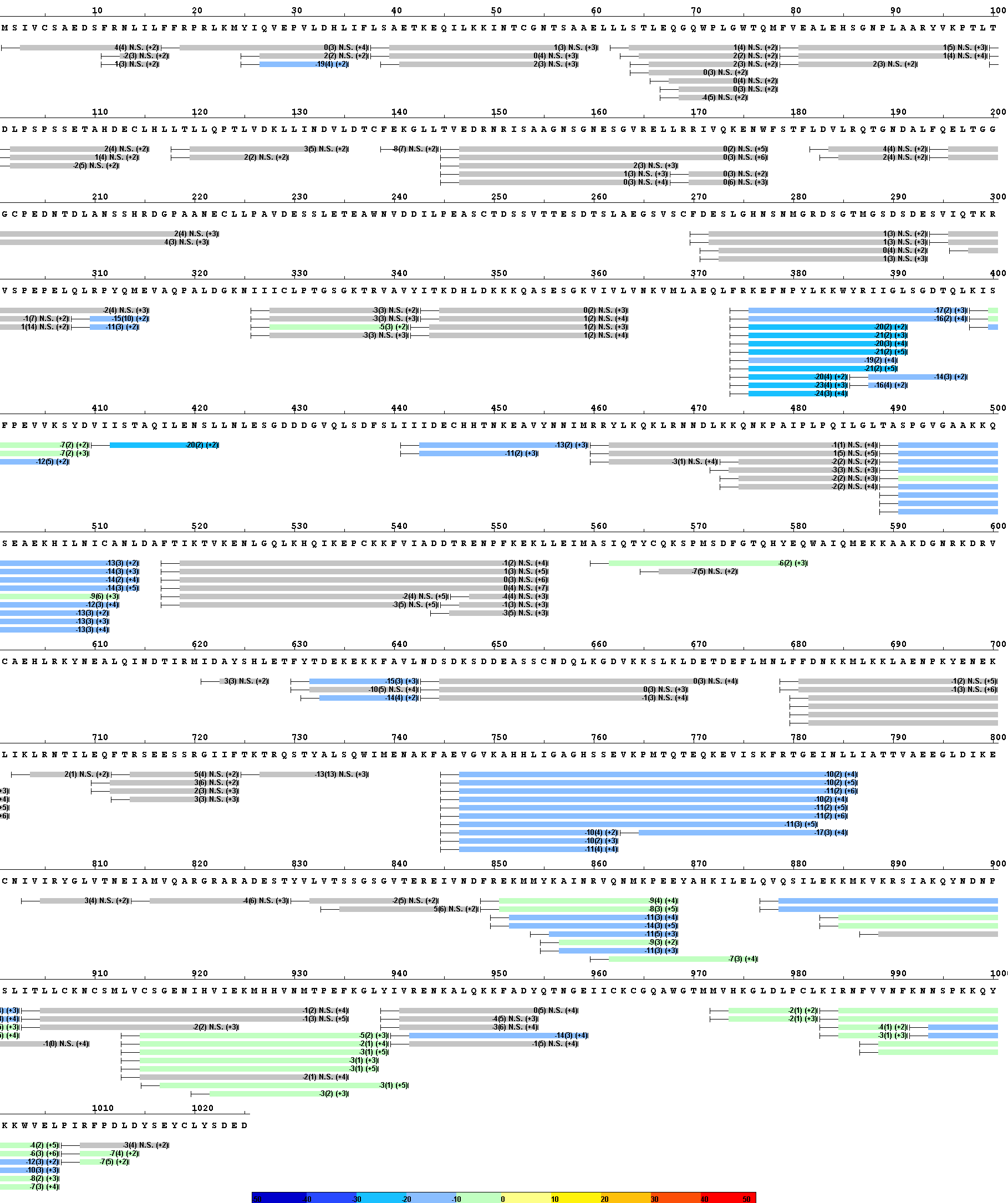
**

**Figure S2b: Differential HDX data for MDA5 ± polyIC&ATP**

**
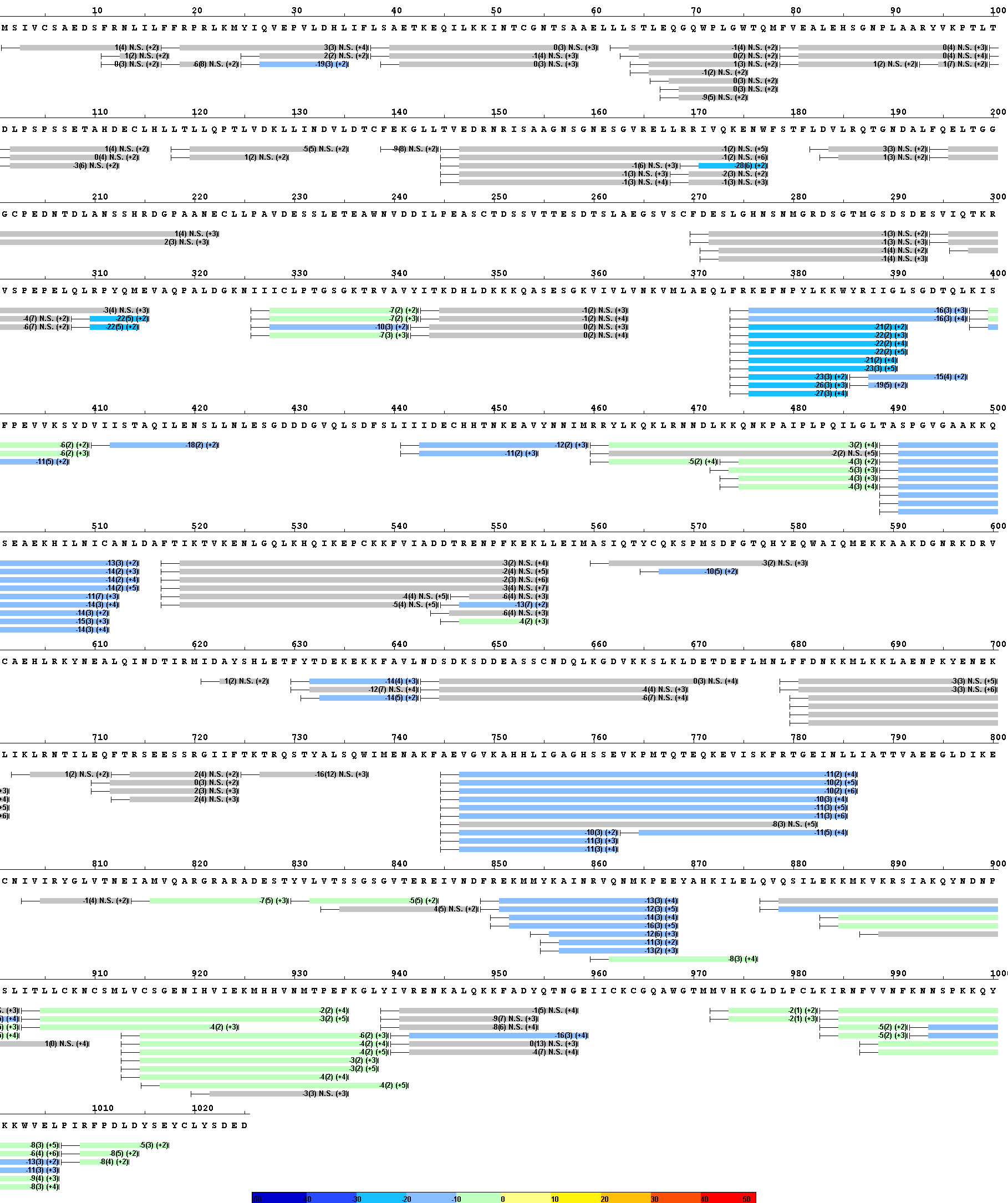
**

**Figure S2c: Differential HDX data for MDA5ΔCARDs ± polyIC**

**
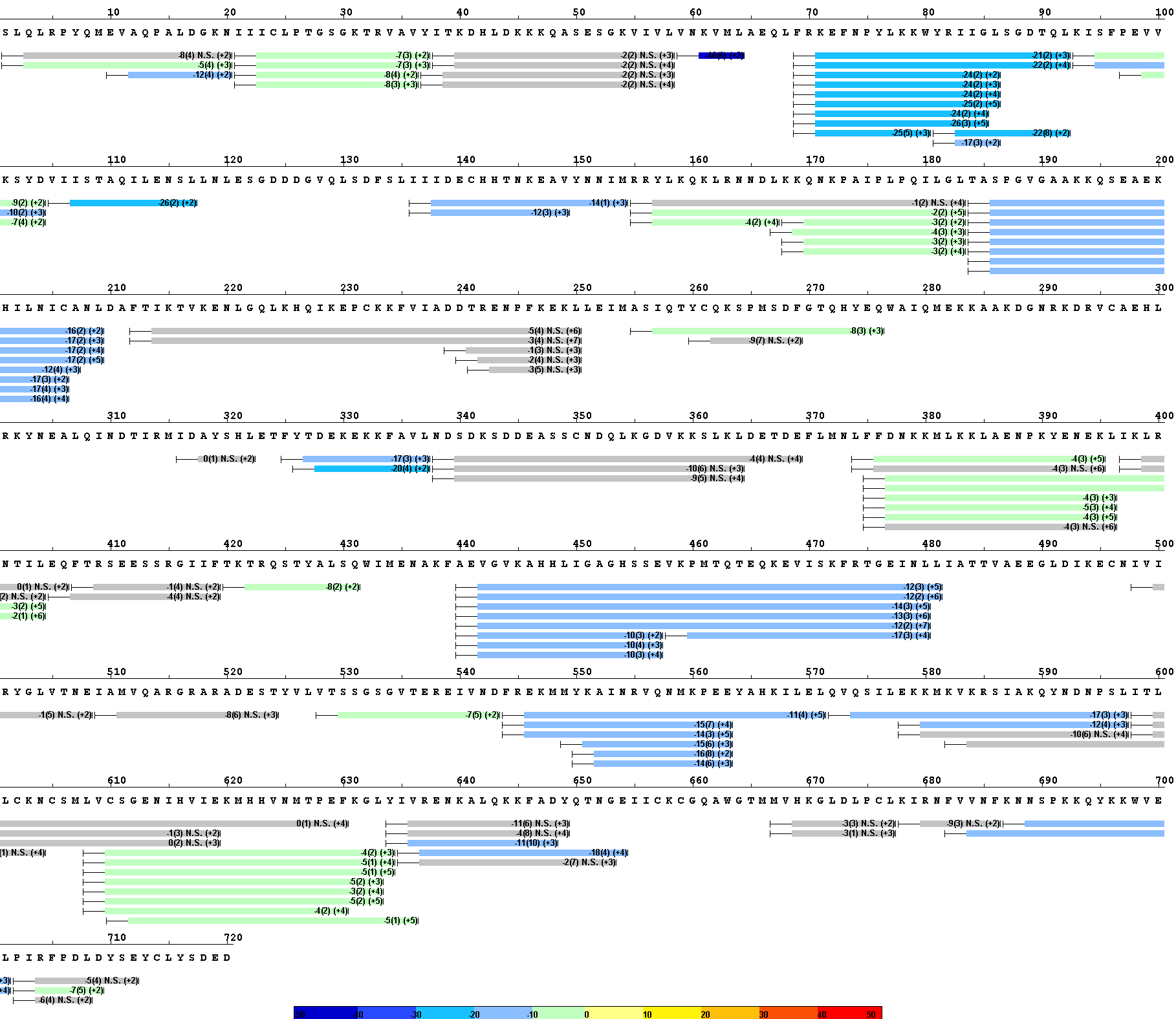
**

**Figure S2d: Differential HDX data for MDA5ΔCARDs ± polyIC&ATP**

**
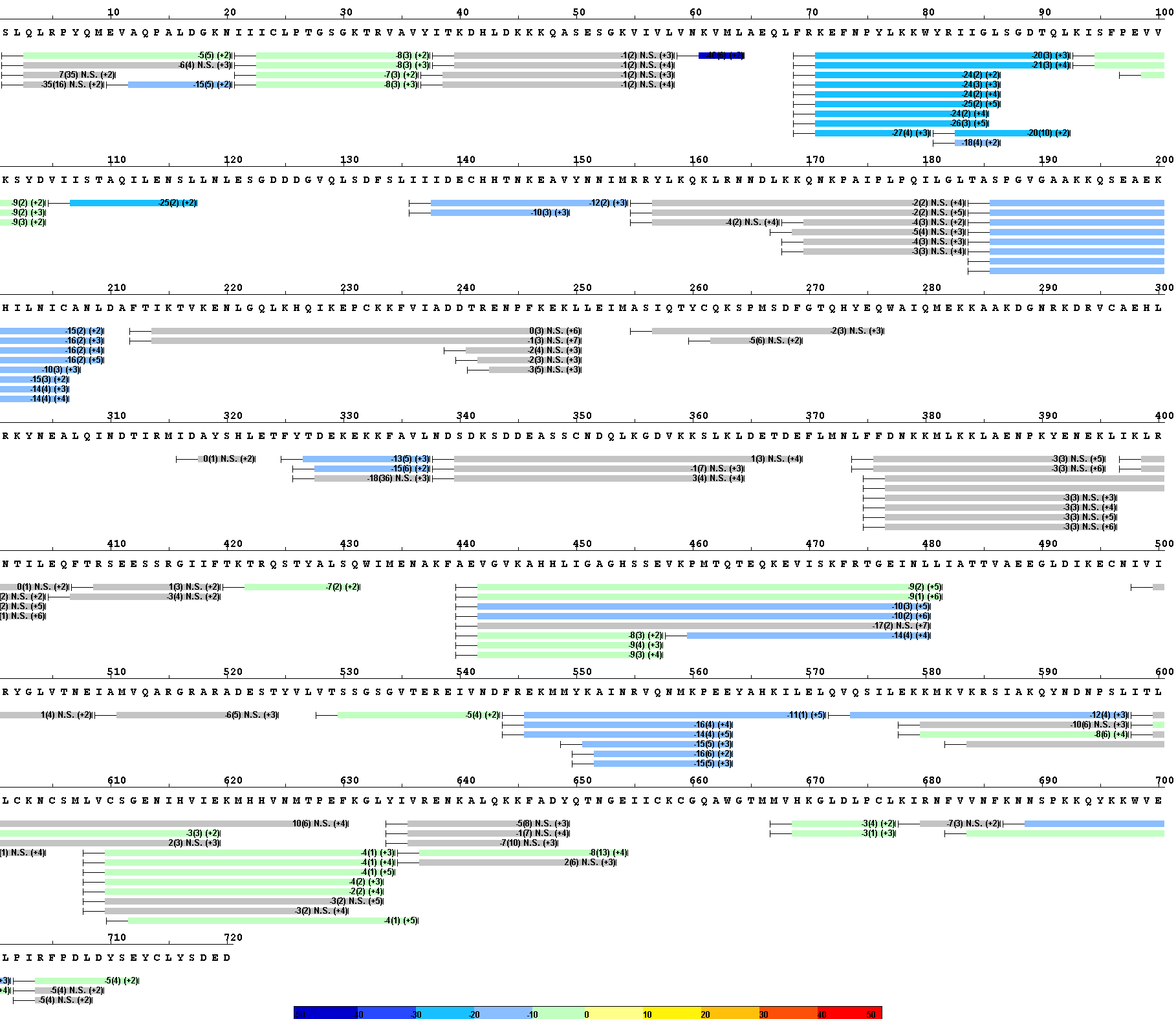
**

**Supplementary figure 3. HDX plots of RIG-I peptides associated with ATP effect**

Deuterium uptake plots of selected RIG-I peptides associated with ATP effects are shown. The data are plotted as percent deuterium uptake versus time on a logarithmic scale. Red, blue and green plots represent RIG-I apoenzyme, + 3p10L and + 3p10L&ATP state, respectively.

**
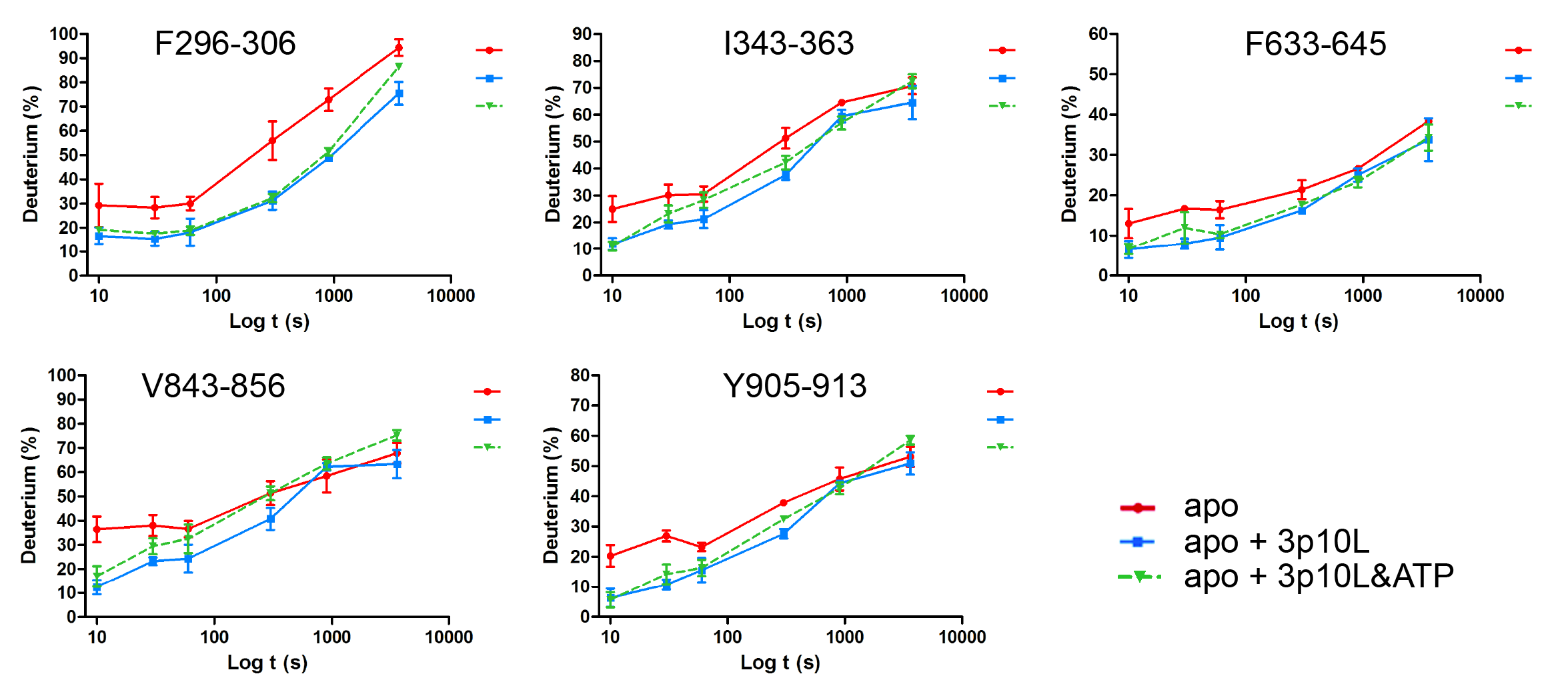
**

**Supplementary references**

Jiang F, Ramanathan A, Miller MT, Tang GQ, Gale M, Jr., Patel SS, Marcotrigiano J (2011) Structural basis of RNA recognition and activation by innate immune receptor RIG-I. *Nature* **479:** 423-427

Kohlway A, Luo D, Rawling DC, Ding SC, Pyle AM (2013) Defining the functional determinants for RNA surveillance by RIG-I. *EMBO Rep* **14:** 772-779

Kowalinski E, Lunardi T, McCarthy AA, Louber J, Brunel J, Grigorov B, Gerlier D, Cusack S (2011) Structural basis for the activation of innate immune pattern-recognition receptor RIG-I by viral RNA. *Cell* **147:** 423-435

Luo D (2014) Toward a crystal-clear view of the viral RNA sensing and response by RIG-I-like receptors. *RNA Biol* **11:** 25-32

Luo D, Ding SC, Vela A, Kohlway A, Lindenbach BD, Pyle AM (2011) Structural insights into RNA recognition by RIG-I. *Cell* **147:** 409-422

Luo D, Kohlway A, Vela A, Pyle AM (2012) Visualizing the determinants of viral RNA recognition by innate immune sensor RIG-I. *Structure* **20:** 1983-1988

Vela A, Fedorova O, Ding SC, Pyle AM (2012) The thermodynamic basis for viral RNA detection by the RIG-I innate immune sensor. *J Biol Chem* **287:** 42564-42573
